# Association of Breastfeeding and Air Pollution Exposure With Lung Function in Chinese Children

**DOI:** 10.1001/jamanetworkopen.2019.4186

**Published:** 2019-05-24

**Authors:** Chuan Zhang, Yuming Guo, Xiang Xiao, Michael S. Bloom, Zhengmin Qian, Craig A. Rolling, Hong Xian, Shao Lin, Shanshan Li, Gongbo Chen, Pasi Jalava, Marjut Roponen, Maija-Riitta Hirvonen, Mika Komppula, Ari Leskinen, Steve Hung Lam Yim, Duo-Hong Chen, Huimin Ma, Xiao-Wen Zeng, Li-Wen Hu, Kang-Kang Liu, Bo-Yi Yang, Guang-Hui Dong

**Affiliations:** 1 Guangdong Provincial Engineering Technology Research Center of Environmental Pollution and Health Risk Assessment, Department of Occupational and Environmental Health, School of Public Health, Sun Yat-sen University, Guangzhou, China; 2 Department of Epidemiology and Preventive Medicine, School of Public Health and Preventive Medicine, Monash University, Melbourne, Victoria, Australia; 3 Department of Environmental Health Sciences, University at Albany, State University of New York, Rensselaer; 4 Department of Epidemiology and Biostatistics, University at Albany, State University of New York, Rensselaer; 5 Department of Epidemiology and Biostatistics, College for Public Health & Social Justice, Saint Louis University, St Louis, Missouri; 6 Department of Environmental and Biological Science, University of Eastern Finland, Kuopio, Finland; 7 Finnish Meteorological Institute, Kuopio, Finland; 8 Department of Applied Physics, University of Eastern Finland, Kuopio, Finland; 9 Department of Geography and Resource Management, Stanley Ho Big Data Decision Analytics Research Centre, Institute of Environment, Energy and Sustainability, The Chinese University of Hong Kong, Shatin, China; 10 Guangdong Environmental Monitoring Center, State Environmental Protection Key Laboratory of Regional Air Quality Monitoring, Guangdong Environmental Protection Key Laboratory of Atmospheric Secondary Pollution, Guangzhou, China; 11 State Key Laboratory of Organic Geochemistry and Guangdong Key Laboratory of Environmental Protection and Resources Utilization, Guangzhou Institute of Geochemistry, Chinese Academy of Sciences, Guangzhou, China

## Abstract

**Importance:**

Breastfeeding and exposure to ambient air pollutants have been found to be independently associated with respiratory health in children; however, previous studies have not examined the association of breastfeeding as a potential moderator of the association.

**Objective:**

To assess associations of breastfeeding and air pollution with lung function in children.

**Design, Setting, and Participants:**

Using a cross-sectional study design, children were recruited from 62 elementary and middle schools located in 7 Chinese cities from April 1, 2012, to October 31, 2013. Data analyses were conducted from November 1, 2018, to March 31, 2019.

**Exposures:**

Long-term concentrations of airborne particulate matter with a diameter of 1 μm or less (PM_1_), airborne particulate matter with a diameter of 2.5 μm or less (PM_2.5_), airborne particulate matter with a diameter of 10 μm or less (PM_10_), and nitrogen dioxide were estimated using a spatial statistical model matched to children’s geocoded home addresses, and concentrations of PM_10_, sulfur dioxide, nitrogen dioxide, and ozone were measured by local air monitoring stations.

**Main Outcomes and Measures:**

Breastfeeding was defined as maternal report of having mainly breastfed for longer than 3 months. Lung function was measured using portable electronic spirometers. Using previously published predicted spirometric values for children in Northeast China as the reference, lung impairment was defined as forced vital capacity (FVC) less than 85%, forced expiratory volume in the first second of expiration less than 85%, peak expiratory flow less than 75%, or maximum midexpiratory flow less than 75%.

**Results:**

Participants included 6740 children (mean [SD] age, 11.6 [2.1] years; 3382 boys [50.2%]). There were 4751 children (70.5%) who were breastfed. Mean (SD) particulate matter concentrations ranged from 46.8 (6.5) μg/m^3^ for PM_1_ to 95.6 (9.8) μg/m^3^ for PM_10_. The prevalence of lung function impairment ranged from 6.8% for peak expiratory flow to 11.3% for FVC. After controlling for age, sex, and other covariates, 1–interquartile range greater concentration of pollutants was associated with higher adjusted odds ratios (AORs) for lung function impairment by FVC among children who were not breastfed compared with those who were (PM_1_: AOR, 2.71 [95% CI, 2.02-3.63] vs 1.20 [95% CI, 0.97-1.48]; PM_2.5_: AOR, 2.27 [95% CI, 1.79-2.88] vs 1.26 [95% CI, 1.04-1.51]; and PM_10_: AOR, 1.93 [95% CI, 1.58-2.37] vs 1.46 [95% CI, 1.23-1.73]). Younger age (<12 years) was associated with lower lung function impairment among the children who had been breastfed. In children from elementary schools, 1–interquartile range greater concentration of pollutants was associated with higher AORs for lung function impairment by FVC among children who had not been breastfed compared with those who had (PM_1_: AOR, 6.43 [95% CI, 3.97-10.44] vs 1.89 [95% CI, 1.28-2.80]; PM_2.5_: AOR, 3.83 [95% CI, 2.63-5.58] vs 1.50 [95% CI, 1.12-2.01]; and PM_10_: AOR, 2.61 [95% CI, 1.90-3.57] vs 1.52 [95% CI, 1.19-1.95]). Results from linear regression models also showed associations of air pollution with worse lung function among children who were not breastfed compared with their counterparts who were breastfed, especially for FVC (PM_1_: β, −240.46 [95% CI, −288.71 to −192.21] vs −38.21 [95% CI, −69.27 to −7.16] mL) and forced expiratory volume in the first second of expiration (PM_1_: β, −201.37 [95% CI, −242.08 to −160.65] vs −30.30 [95% CI, −57.66 to −2.94] mL).

**Conclusions and Relevance:**

In this study, breastfeeding was associated with lower risk of lung function impairment among children in China exposed to air pollution, particularly among younger children.

## Key Points

**Question:**

Is having been breastfed associated with lower risk of impaired lung function in children exposed to ambient air pollution?

**Findings:**

In this cross-sectional study of 6740 Chinese children aged 7 to 14 years, exposure to greater concentrations of particulate matter air pollution was associated with higher rates of lung function impairment among children who had not been breastfed compared with those who had been breastfed.

**Meaning:**

These findings suggest that breastfeeding is associated with lower risk of lung function impairment among children exposed to ambient air pollutants.

## Teaser

This cross-sectional study evaluates the association of air pollution exposure and having been breastfed or not with lung function among children in China.

## Introduction

Many studies have reported that ambient air pollution and breastfeeding are associated with a variety of respiratory health outcomes in children, including asthma, pneumonia, bronchitis, acute lower respiratory tract infections, and impaired lung function development.^1,2,3,4,5,6^ However, most respiratory data in the literature were captured using questionnaires, which are vulnerable to recall bias and misclassification. Although more challenging to implement in large population-based studies, direct lung function measures using spirometry offer an objective quantification of respiratory health. A 2008 study by Götschi et al^7^ reviewed 58 publications investigating the long-term associations of air pollution exposure with lung function and found that 50 articles (86%) reported a statistically significant adverse association of air pollution exposure with lung function.

Substantial evidence suggests that breastfeeding has a favorable association with lung function in children^5,8,9,10^ as well as a mitigating effect on the adverse effects of other environmental factors, including ambient air pollution and environmental tobacco smoke.^3,11,12^ A 2006 birth cohort study from the Czech Republic^11^ suggested that the association of coal home heating and maternal smoking with the incidence of lower respiratory illness varied with breastfeeding status.

However, few studies have examined the associations of both breastfeeding and air pollution with lung function, to our knowledge; thus, the objective of this study was to assess the association of breastfeeding and air pollution with lung function in children. We hypothesized that breastfeeding may moderate the association of air pollution with lung function. Children experience body growth and development and are more sensitive to environmental factors than adults, which could help in understanding the association of exposure to air pollution with breastfeeding. Therefore, using data from the Seven Northeastern Cities study,^13^ we assessed the association of breastfeeding and estimated exposure to air pollution with lung function in 6740 children.

## Methods

### Study Population

Using a cross-sectional study design, the Seven Northeastern Cities study^13^ investigated associations of various health outcomes with ambient air pollution among school children from April 1, 2012, to October 31, 2013. The study population has been described previously.^6^ Briefly, we chose study sites in 7 Liaoning province cities in the northeast of China to maximize pollution gradients based on 2009 to 2011 monitoring data. We selected 24 urban districts among the 7 study cities of Shenyang, Dalian, Fushun, Anshan, Benxi, Liaoyang, and Dandong. In each district, we randomly selected 1 or 2 elementary schools and 1 or 2 middle schools located within 2 km of a municipal monitoring station, for a total of 62 schools. Within each school, we randomly selected 1 or 2 classes from each grade level, depending on the class size, to enroll study participants. Detailed information on the random sampling procedures are described in eMethods 1 in the Supplement. Children were included if they lived in the district for at least 2 years before the start of the study.

After obtaining permission from each school principal, we provided classroom teachers with information packets containing a description of the study and a study questionnaire to distribute to students’ parents or guardians. We were careful to emphasize the noncompulsory nature of participation to the teachers to ensure only voluntary participation by parents. Then teachers obtained written consent from parents or guardians and invited consenting parents and guardians to an information session. Parents or guardians completed the study questionnaires at the time of the information session or at home, in which case the student delivered the completed document to the classroom teacher.

Analysis was conducted from November 1, 2018, to March 31, 2019. We followed the Strengthening the Reporting of Observational Studies in Epidemiology (STROBE) reporting guideline for cross-sectional studies. The study protocol was approved by the Human Studies Committee of Sun Yat-sen University, Guangzhou, China. Written informed assent from each participating child and written informed consent from their parent or guardian were obtained in advance of the study.

### Covariates

We considered 16 covariates a priori based on evidence in the literature^1,2,3,4,5,6,7,8,9,10,11,12,13^ for associations with air pollution exposure and respiratory function: age (years), sex (male or female), height (centimeters), birth weight (kilograms), preterm birth (yes or no), parental education (≥high school or lower), annual family income (<¥5000 [<US $744.11], ¥5000-¥9999 [US $744.11-$1488.08], ¥10 000-¥29 999 [US $1488.23-$4464.54], ¥30 000-¥99 999 [US $4464.68-$14 882.13], or >¥100 000 [>US $14 882.28]), exercise per week (hours), passive smoke exposure (yes or no), home coal use (yes or no), presence of a house pet (yes or no), home renovation in the past 2 years (yes or no), area of residence per person (meters^2^), asthma diagnosed by a physician (yes or no), family history of atopy (yes or no), and short-term air pollution concentrations (micrograms per meter^3^). A detailed description of these covariates is listed in eMethods 2 in the Supplement.

We defined breastfeeding as having been mainly breastfed for longer than 3 months.^3^ The mothers of children participating in this study received 3 months of maternity leave from their jobs after delivery. After 3 months, most mothers discontinued breastfeeding to return to the workplace. Mainly breastfed indicates that the child was fed mainly by breast milk and occasionally fed by animal milk. The early diet of children not breastfed included animal milk, juice, and soups made from egg, rice, chicken, pork, beef, fish, or vegetables.^3^

### Pulmonary Function Measurement (Spirometry)

Our procedures for lung function measurement (spirometry) and data management have previously been described in detail.^6,14^ Briefly, 2 of us (G.-H.D. and L.-W.H.) measured forced vital capacity (FVC), forced expiratory volume in the first second of expiration (FEV_1_), peak expiratory flow (PEF), and maximum midexpiratory flow according to a standardized procedure using 2 SpiroLab portable electronic spirometers (Medical International Research).^15^ Children were tested in the standing position, wearing a nose clip, and in a quiet and comfortable room.^16,17^ Using previously published predicted spirometric values for children in northeast China as the reference,^18^ we defined impaired lung function as FVC less than 85% of reference, FEV_1_ less than 85% of reference, maximum midexpiratory flow less than 75% of reference, or PEF less than 75% of reference.

### Ambient Air Pollution

We estimated daily airborne particulate matter with a diameter of 1 μm or less (PM_1_), airborne particulate matter with a diameter of 2.5 μm or less (PM_2.5_), airborne particulate matter with a diameter of 10 μm or less (PM_10_), and nitrogen dioxide (NO_2_) concentrations with a machine learning method at a spatial resolution of 10 km by 10 km from January 1, 2009, through December 31, 2012, based on satellite remote sensing, meteorological data, and land use information. The results of a 10-fold cross-validation showed coefficient of determination values for daily predictions were 55% for PM_1_ and 86% for PM_2.5_, and coefficient of determination values for annual predictions were 75% for PM_1_ and 86% for PM_2.5_. The root-mean-square error values for daily predictions were 12.4 μg/m^3^ for NO_2_ and 31.5 μg/m^3^ for PM_10_, and the root-mean-square error values for annual predictions were 6.5 μg/m^3^ for NO_2_ and 14.4 μg/m^3^ for PM_10_. Detailed information regarding the analysis has been published elsewhere^19,20^ and is presented in eMethods 3 in the Supplement. Other concentrations of pollutants were measured at municipal air monitoring stations in each study district, as described previously^3,13,21^ and in eMethods 4 in the Supplement. Four-year mean air pollutant concentrations from 2009 to 2012 were calculated and assigned to each child as surrogates for long-term exposure.

### Statistical Analysis

We assessed normality and described distributions for continuous variables as the mean and SD and categorical variables as proportions comparing children who were breastfed with children who were not breastfed by *t* test or χ^2^ test, as appropriate. To investigate associations of results of dichotomized pulmonary function tests with ambient air pollution, we used a 2-level logistic regression model in which children were the first-level units and study districts were the second-level units. The details of this model are described in eMethods 5 in the Supplement. We also used multiple linear mixed-effects models to estimate associations of continuous lung function measures with ambient air pollutants. We evaluated the differences according to breastfed status by including the association of this variable with air pollutants in the regression models. To evaluate the robustness of our estimates, we conducted sensitivity analyses, including stratifying by child’s age, excluding children with mixed feeding, excluding children with low birth weight or preterm birth, excluding children with an asthma diagnosis from a physician, and excluding regions 1 at a time. We also adjusted for the covariates listed previously. If the estimated regression for air pollutants changed by more than 10% on inclusion in the base model, the covariate was retained in the final model as a confounding variable.

Analyses were conducted using GLIMMIX in SAS statistical software version 9.4 (SAS Institute). Statistical tests were 2-tailed. *P* values less than .05 were considered statistically significant for main effects, and *P* values less than .10 were considered statistically significant for interactions. For effect size estimates, 95% CIs were calculated.

## Results

### Characteristics of the Study Population

The parents of 7326 children were invited to participate, and 7109 children (97.0%) completed spirometry. We excluded 279 children (3.9%) who had lived in their district for less than 2 years and 90 children (1.3%) who lacked necessary information, such as age and sex. This left 6740 children aged 7 to 14 years (mean [SD] age, 11.6 [2.1] years; 3382 boys [50.2%]) from 24 study districts in the analysis. Characteristics of the children included in the study are presented in Table 1. Baseline characteristics were similar among children included in and excluded from analysis and are presented in eTable 1 in the Supplement.

**Table 1.** Characteristics of Children Included in Study

Characteristic	No. (%)		
	Not Breastfed (n = 1989)	Breastfed (n = 4751)	Total (N = 6740)
Age, mean (SD), y^a^	11.4 (2.1)	11.6 (2.1)	11.6 (2.1)
Height, mean (SD), cm^a^	153.4 (12.9)	154.2 (12.5)	153.9 (12.6)
Weight, mean (SD), kg	47.9 (15.6)	48.7 (15.6)	48.4 (15.6)
Body mass index, mean (SD)^b^	19.9 (4.3)	20.1 (4.8)	20.0 (4.7)
Birth weight, mean (SD), kg^a^	3.37 (0.49)	3.41 (0.47)	3.40 (0.48)
Area of residence per person, mean (SD), m^2^^a^	23.6 (9.9)	22.4 (9.7)	22.7 (9.8)
Exercise, mean (SD), h/wk^a^	7.3 (6.9)	7.7 (8.1)	7.6 (7.7)
Boys^a^	1070 (53.8)	2312 (48.7)	3382 (50.2)
Preterm birth^a^	150 (7.5)	188 (4.0)	338 (5.0)
Parental education ≥high school	1264 (63.6)	2947 (62.0)	4211 (62.5)
Family income per year, ¥^a^,			
<5000^c^	232 (11.7)	526 (11.1)	758 (11.2)
5000-9999^d^	255 (12.8)	621 (13.1)	876 (13.0)
10 000-29 999^e^	644 (32.4)	1750 (36.8)	2394 (35.5)
30 000-99 999^f^	758 (38.1)	1679 (35.3)	2437 (36.2)
>100 000^g^	100 (5.0)	175 (3.7)	275 (4.1)
Passive smoke exposure			
Father	590 (29.7)	1532 (32.3)	2122 (31.5)
Mother	186 (9.4)	422 (8.9)	608 (9.0)
Other	175 (8.8)	376 (7.9)	551 (8.2)
Anyone	951 (47.8)	2330 (49.0)	3281 (48.7)
Maternal smoking during pregnancy	15 (0.8)	39 (0.8)	54 (0.8)
Home coal use^a^	170 (8.6)	506 (10.7)	676 (10.0)
Pet kept in home	452 (22.7)	983 (20.7)	1435 (21.3)
Home renovation in past 2 y	706 (35.5)	1710 (36.0)	2416 (35.9)
Family history of atopy	414 (20.8)	976 (20.5)	1390 (20.6)
Asthma diagnosis^a^	160 (8.0)	300 (6.3)	460 (6.8)
Current asthma	102 (5.1)	195 (4.1)	297 (4.4)
Spirometric parameters, mean (SD)^a^			
FVC, L	2.6 (0.8)	2.6 (0.7)	2.6 (0.8)
FEV_1_, L	2.4 (0.7)	2.5 (0.7)	2.5 (0.7)
PEF, L/s	4.7 (1.5)	4.8 (1.4)	4.8 (1.4)
MMEF, L/s	3.3 (1.1)	3.4 (1.0)	3.4 (1.1)
Lung function status^a^			
FVC <85% of predicted value	269 (13.5)	490 (10.3)	759 (11.3)
FEV_1_ <85% of predicted value	215 (10.8)	363 (7.6)	578 (8.6)
PEF <75% of predicted value	166 (8.4)	292 (6.2)	458 (6.8)
MMEF <75% of predicted value	216 (10.9)	418 (8.8)	634 (9.4)

Abbreviations: FEV_1_, forced expiratory volume in the first second of expiration; FVC, forced vital capacity; MMEF, maximum mid expiratory flow rate; PEF, peak expiratory flow rate.

^a^ Significant difference exists between children who were breastfed and those who were not as tested by χ^2^ test for categorical variables and *t* test for continuous variables (*P* < .05).

^b^ Calculated as weight in kilograms divided by height in meters squared.

^c^ Less than US $744.11.

^d^ US $744.11 to US $1488.08.

^e^ US $1488.23 to US $4464.54.

^f^ US $4464.68 to US $14 882.13.

^g^ Greater than US $14 882.28.

There were 4751 children (70.5%) who were breastfed. The difference in spirometric measures and lung function status was statistically significant when stratified by breastfed status. Children who were breastfed had greater spirometric measure values and a lower prevalence of impaired lung function compared with children who were not breastfed. The distributions of air pollutants, lung function tests, and breastfeeding prevalence are shown in eTable 2 in the Supplement for each city.

### Ambient Air Pollutant Concentrations

Table 2 presents the estimated annual mean air pollutant concentrations and their pairwise correlations. The 4-year mean (SD) 24-hour PM_1_, PM_2.5_, PM_10_, and NO_2_ concentrations estimated using a spatial statistical model were 46.8 (6.5) μg/m^3^, 54.0 (6.1) μg/m^3^, 95.6 (9.8) μg/m^3^, and 33.6 (4.7) μg/m^3^, respectively. Daily mean air pollutant levels during lung function measurement in the children are shown in eTable 3 in the Supplement.

**Table 2.** Distribution of 4-Year Mean Air Pollutant Concentrations and Pairwise Correlations

Pollutant	Annual Concentration, μg/m^3^				Spearman Correlation Coefficient							
					Based on a Spatial Statistical Model				Based on Air Monitoring Station			
	Mean (SD)	Median (IQR)	1-IQR Range	>WHO AQG, %^a^	PM_1_	PM_2.5_	PM_10_	NO_2_	PM_10_	SO_2_	NO_2_	O_3_
Air pollutants estimated using a spatial statistical model												
PM_1_	46.8 (6.5)	45.2 (41.0-54.1)	13.1	NA^b^	1	0.93^c^	0.87^c^	0.73^c^	0.55^c^	0.29^c^	0.03	0.36^c^
PM_2.5_	54.0 (6.1)	52.1 (48.8-58.8)	10.0	100	NA	1	0.97^c^	0.85^c^	0.49^c^	0.33^c^	0.13	0.35^c^
PM_10_	95.6 (9.8)	94.6 (89.3-103.1)	13.8	100	NA	NA	1	0.92^c^	0.48^c^	0.35^c^	0.15	0.36^c^
NO_2_	33.6 (4.7)	32.3 (31.2-38.5)	7.3	7.9	NA	NA	NA	1	0.48^c^	0.26	0.14	0.35^c^
Measured by local air monitoring stations												
PM_10_	88.9 (21.3)	90.4 (74.4-105.0)	30.6	100	NA	NA	NA	NA	1	0.58^c^	0.47^c^	0.85^c^
SO_2_	49.8 (16.0)	48.4 (38.1-61.5)	23.4	95.8	NA	NA	NA	NA	NA	1	0.21	0.60^c^
NO_2_	36.4 (11.1)	35 (31.8-44.8)	13	37.5	NA	NA	NA	NA	NA	NA	1	0.33
O_3_	106.9 (165.8)	43.8 (30.7-77)	46.3	12.5	NA	NA	NA	NA	NA	NA	NA	1

Abbreviations: IQR, interquartile range; NA, not applicable; NO_2_, nitrogen dioxide; O_3_, ozone; PM_1_, particle matter with aerodynamic diameter ≤1.0 μm; PM_2.5_, particle matter with aerodynamic diameter ≤2.5 μm; PM_10_, particle matter with aerodynamic diameter ≤10 μm; SO_2_, sulfur dioxide; WHO AQG, World Health Organization air quality guidelines.

^a^ Compared with the WHO AQG (the WHO AQG for PM_2.5_, PM_10_, SO_2_, NO_2_, and O_3_ were 10 μg/m^3^, 20 μg/m^3^, 20 μg/m^3^, 40 μg/m^3^, and 400 μg/m^3^, respectively).

^b^ A guideline for PM_1_ has not been proposed by WHO or any other organization.

^c^ *P* < .05.

### Associations of Air Pollutants With Lung Function

Crude association estimates of air pollution with lung function are presented in eTable 4 and eTable 5 in the Supplement. The prevalence of lung function impairment ranged from 6.8% for peak expiratory flow to 11.3% for FVC. When stratified by breastfed status, we observed associations of PM_1_ and PM_2.5_ exposure with the prevalence of impaired percent of FVC and percent of FEV_1_ in children. These linear trend associations were more pronounced in children who were not breastfed than in children who were breastfed (eFigure 1 in the Supplement). Table 3 presents the adjusted odds ratios (AORs) for children’s lung function impairment using 4-year air pollutant concentrations (eg, PM_1_) as predictors while adjusting for the other covariates. Higher odds of lung function impairment were associated with greater air pollutant concentrations, and the odds were lower among children who were breastfed than among children who were not breastfed. One–interquartile range (IQR) greater concentration of pollutants was associated with higher AORs for impaired FVC among children who were not breastfed compared with those who were (PM_1_: AOR, 2.71 [95% CI, 2.02-3.63] vs 1.20 [95% CI, 0.97-1.48]; PM_2.5_: AOR, 2.27 [95% CI, 1.79-2.88] vs 1.26 [95% CI, 1.04-1.51]; PM_10_: AOR, 1.93 [95% CI, 1.58-2.37] vs 1.46 [95% CI, 1.23-1.73]). Interactions measuring heterogeneity of the associations of air pollution concentrations with lung function impairment by breastfed status were statistically significant.

**Table 3.** Adjusted Odds Ratios of Impaired Lung Function and 4-Year Mean Ambient Air Pollutant Concentrations Estimated Using a Spatial Statistical Model Stratified by Breastfed Status

Pollutant	AOR (95% CI)^a^^,^^b^		*P* Value for Interaction
	Not Breastfed (n = 1989)	Breastfed (n = 4751)	
FVC <85% of predicted value			
PM_1_	2.71 (2.02-3.63)	1.20 (0.97-1.48)	<.001
PM_2.5_	2.27 (1.79-2.88)	1.26 (1.04-1.51)	<.001
PM_10_	1.93 (1.58-2.37)	1.46 (1.23-1.73)	.02
NO_2_	1.87 (1.49-2.36)	1.69 (1.39-2.04)	.45
FEV_1_ <85% of predicted value			
PM_1_	2.81 (2.02-3.90)	1.46 (1.14-1.87)	.001
PM_2.5_	2.64 (2.01-3.47)	1.55 (1.25-1.92)	.001
PM_10_	2.43 (1.93-3.06)	1.81 (1.49-2.20)	.03
NO_2_	2.58 (1.98-3.36)	2.20 (1.76-2.75)	.31
PEF <75% of predicted value			
PM_1_	1.74 (1.24-2.45)	1.27 (0.97-1.66)	.12
PM_2.5_	1.56 (1.18-2.08)	1.21 (0.96-1.52)	.14
PM_10_	1.44 (1.12-1.84)	1.21 (0.98-1.50)	.25
NO_2_	1.49 (1.13-1.96)	1.32 (1.05-1.67)	.47
MMEF <75% of predicted value			
PM_1_	1.43 (1.06-1.94)	1.23 (0.98-1.54)	.41
PM_2.5_	1.37 (1.06-1.75)	1.24 (1.02-1.51)	.53
PM_10_	1.34 (1.08-1.67)	1.29 (1.08-1.55)	.78
NO_2_	1.37 (1.07-1.75)	1.40 (1.14-1.72)	.88

Abbreviations: AOR, adjusted odds ratio; FEV_1_, forced expiratory volume in the first second of expiration; FVC, forced vital capacity; MMEF, maximum midexpiratory flow rate; NO_2_, nitrogen dioxide; PEF, peak expiratory flow rate; PM_1_, airborne particulate matter with a diameter ≤1 μm; PM_2.5_, airborne particulate matter with a diameter ≤2.5 μm; PM_10_, airborne particulate matter with a diameter ≤10 μm.

^a^ Adjusted for age, sex, height, birth weight, preterm birth, parental education, annual family income, exercise per week, passive smoke exposure, home coal use, presence of a house pet, home renovation in the past 2 years, area of residence per person, asthma diagnosis, family history of atopy, and short-term air pollution concentrations.

^b^ Effect expressed for 1–interquartile range change in ambient concentration for each pollutant (PM_1_, 13.1 μg/m^3^; PM_2.5_, 10.0 μg/m^3^; PM_10_, 13.8 μg/m^3^; and NO_2_, 7.3 μg/m^3^).

Table 4 presents the association of continuous lung function measures with air pollutants. Consistent with the results for dichotomous lung function impairment, we observed negative associations of air pollution exposure with lung function. Among children who were not breastfed, for each IQR increase in pollutant concentration, FEV_1_ was 201.37 mL lower (95% CI, −242.08 to −160.65) for PM_1_, 181.68 mL lower (95% CI, −217.06 to −146.30) for PM_2.5_, and 154.14 mL lower (95% CI, −186.37 to −121.90) for PM_10_. Among children who were breastfed, FEV_1_ was 30.30 mL lower (95% CI, −57.66 to −2.94) for PM_1_, 33.86 mL lower (95% CI, −57.57 to −10.15) for PM_2.5_, and 48.63 mL lower (95% CI, −70.77 to −26.49) for PM_10_ for each IQR increase in concentrations. Results from linear regression models also showed associations of air pollution with worse lung function among children who were not breastfed compared with their counterparts who were breastfed, especially for FVC (PM_1_: β, −240.46 [95% CI, −288.71 to −192.21] vs −38.21 [95% CI, −69.27 to −7.16] mL) and forced expiratory volume in the first second of expiration (PM_1_: β, −201.37 [95% CI, −242.08 to −160.65] vs −30.30 [95% CI, −57.66 to −2.94] mL). The associations of breastfeeding and air pollutants measured from routine monitoring stations with lung function in children are shown in eTable 6 and eTable 7 in the Supplement.

**Table 4.** Estimated Absolute Change in Children’s Lung Function Test Measurements Associated With 4-Year Mean Ambient Air Pollutant Concentrations Estimated Using a Spatial Statistical Model Stratified by Breastfed Status

Pollutant	β (95% CI)^a^^,^^b^		*P* Value for Interaction
	Not Breastfed (n = 1989)	Breastfed (n = 4751)	
FVC			
PM_1_	−240.46 (−288.71 to −192.21)	−38.21 (−69.27 to −7.16)	<.001
PM_2.5_	−217.97 (−259.95 to −175.99)	−42.02 (−68.78 to −15.27)	<.001
PM_10_	−185.99 (−224.08 to −147.89)	−56.48 (−81.29 to −31.68)	.003
NO_2_	−179.26 (−221.32 to −137.20)	−88.91 (−116.93 to −60.88)	.42
FEV_1_			
PM_1_	−201.37 (−242.08 to −160.65)	−30.30 (−57.66 to −2.94)	<.001
PM_2.5_	−181.68 (−217.06 to −146.30)	−33.86 (−57.57 to −10.15)	<.001
PM_10_	−154.14 (−186.37 to −121.90)	−48.63 (−70.77 to −26.49)	<.001
NO_2_	−150.26 (−186.14 to −114.39)	−79.99 (−104.91 to −55.06)	.16
PEF			
PM_1_	−333.29 (−438.21 to −228.37)	−58.28 (−125.54 to 8.98)	<.001
PM_2.5_	−273.82 (−363.94 to −183.69)	−65.96 (−124.14 to −7.78)	.001
PM_10_	−214.86 (−295.23 to −134.48)	−79.68 (−133.81 to −25.55)	.009
NO_2_	−206.06 (−294.94 to −117.18)	−96.96 (−157.95 to −35.98)	.06
MMEF			
PM_1_	−127.42 (−208.01 to −46.84)	−13.12 (−65.24 to 39.00)	.03
PM_2.5_	−101.86 (−171.22 to −32.49)	−23.95 (−69.18 to 21.28)	.06
PM_10_	−77.17 (−139.14 to −15.19)	−38.00 (−80.34 to 4.34)	.19
NO_2_	−66.16 (−134.19 to 1.87)	−53.38 (−101.08 to −5.69)	.57

Abbreviations: FEV_1_, forced expiratory volume in the first second of expiration; FVC, forced vital capacity; MMEF, maximum midexpiratory flow rate; NO_2_, nitrogen dioxide; PEF, peak expiratory flow rate; PM_1_, airborne particulate matter with a diameter ≤1 μm; PM_2.5_, airborne particulate matter with a diameter ≤2.5 μm; PM_10_, airborne particulate matter with a diameter ≤10 μm.

^a^ Adjusted for age, sex, height, birth weight, preterm birth, parental education, annual family income, exercise per week, passive smoke exposure, home coal use, presence of a house pet, home renovation in the past 2 years, area of residence per person, asthma diagnosis, family history of atopy, and short-term air pollution concentrations.

^b^ Effect expressed for a 1–interquartile range change in ambient concentration for each pollutant (PM_1_, 13.1 μg/m^3^; PM_2.5_, 10.0 μg/m^3^; PM_10_, 13.8 μg/m^3^; and NO_2_, 7.3 μg/m^3^).

### Sensitivity Analyses

The heterogeneity of associations by breastfeeding appeared to be more substantial in younger children (from elementary school) than in older children (Table 5; eFigure 2 in the Supplement). In children from elementary schools, 1–interquartile range greater concentration of pollutants was associated with higher AORs for lung function impairment by FVC among children who had not been breastfed compared with those who had (PM_1_: AOR, 6.43 [95% CI, 3.97-10.44] vs 1.89 [95% CI, 1.28-2.80]; PM_2.5_: AOR, 3.83 [95% CI, 2.63-5.58] vs 1.50 [95% CI, 1.12-2.01]; and PM_10_: AOR, 2.61 [95% CI, 1.90-3.57] vs 1.52 [95% CI, 1.19-1.95]). We found similar results to our main analysis when we excluded children with mixed feeding (n = 302) (eTable 8 and eTable 9 in the Supplement), children with low birth weight (n = 208) or preterm birth (n = 338) (eTable 10 and eTable 11 in the Supplement), or children with asthma diagnosed by a physician (n = 460) (eTable 12 and eTable 13 in the Supplement) during sensitivity analysis. Results were also similar when we excluded study districts 1 at a time (eTable 14 in the Supplement) during sensitivity analyses.

**Table 5.** Adjusted Odds Ratios for Children’s Impaired Lung Function and Mean Ambient Air Pollutant Concentrations by Breastfed and School Status

Pollutant	AOR (95% CI)^a^^,^^b^		*P* Value for Interaction
	Not Breastfed (n = 1181)	Breastfed (n = 2569)	
**Children From Elementary Schools (n = 3750)**			
FVC			
PM_1_	6.43 (3.97-10.44)	1.89 (1.28-2.80)	<.001
PM_2.5_	3.83 (2.63-5.58)	1.50 (1.12-2.01)	.001
PM_10_	2.61 (1.90-3.57)	1.52 (1.19-1.95)	.006
NO_2_	2.32 (1.66-3.25)	1.71 (1.28-2.29)	.15
FEV_1_			
PM_1_	9.26 (5.32-16.13)	2.27 (1.44-3.56)	<.001
PM_2.5_	5.99 (3.85-9.31)	1.72 (1.23-2.42)	<.001
PM_10_	4.08 (2.79-5.96)	1.80 (1.35-2.41)	<.001
NO_2_	3.62 (2.43-5.39)	2.34 (1.67-3.27)	.07
PEF			
PM_1_	1.74 (1.11-2.73)	1.38 (0.93-2.04)	.42
PM_2.5_	1.55 (1.07-2.24)	1.26 (0.93-1.71)	.34
PM_10_	1.43 (1.04-1.98)	1.24 (0.96-1.61)	.43
NO_2_	1.40 (0.99-1.98)	1.30 (0.97-1.75)	.72
MMEF			
PM_1_	1.95 (1.34-2.84)	1.87 (1.18-2.97)	.89
PM_2.5_	1.72 (1.19-2.50)	1.62 (1.21-2.16)	.78
PM_10_	1.67 (1.21-2.29)	1.54 (1.20-1.97)	.66
NO_2_	1.65 (1.17-2.34)	1.63 (1.23-2.17)	.94
**Children From Middle Schools (n = 2990)**			
FVC			
PM_1_	2.48 (1.34-4.56)	0.83 (0.56-1.22)	.003
PM_2.5_	2.87 (1.72-4.80)	1.05 (0.73-1.52)	.001
PM_10_	2.75 (1.78-4.23)	1.65 (1.14-2.39)	.06
NO_2_	2.92 (1.81-4.74)	2.06 (1.40-3.05)	.25
FEV_1_			
PM_1_	2.27 (1.15-4.49)	1.26 (0.80-1.97)	.15
PM_2.5_	3.05 (1.70-5.49)	1.45 (0.95-2.20)	.04
PM_10_	3.09 (1.89-5.05)	1.90 (1.26-2.84)	.12
NO_2_	3.38 (1.97-5.82)	2.08 (1.37-3.16)	.15
PEF			
PM_1_	1.48 (0.95-2.29)	1.44 (0.76-2.73)	.95
PM_2.5_	1.46 (0.97-2.19)	1.38 (0.82-2.32)	.86
PM_10_	1.38 (0.93-2.04)	1.31 (0.84-2.04)	.86
NO_2_	1.33 (0.80-2.21)	1.30 (0.86-1.98)	.95
MMEF			
PM_1_	1.44 (0.83-2.52)	1.37 (0.93-2.01)	.87
PM_2.5_	1.26 (0.89-1.79)	1.18 (0.75-1.85)	.80
PM_10_	1.21 (0.86-1.69)	1.06 (0.72-1.57)	.60
NO_2_	1.18 (0.82-1.70)	0.98 (0.63-1.55)	.51

Abbreviations: AOR, adjusted odds ratio; FEV_1_, forced expiratory volume in the first second of expiration; FVC, forced vital capacity; MMEF, maximum mid expiratory flow rate; NO_2_, nitrogen dioxide; PEF, peak expiratory flow rate; PM_1_, airborne particulate matter with a diameter ≤1 μm; PM_2.5_, airborne particulate matter with a diameter ≤2.5 μm; PM_10_, airborne particulate matter with a diameter ≤10 μm.

^a^ Adjusted for age, sex, height, birth weight, preterm birth, parental education, annual family income, exercise per week, passive smoke exposure, home coal use, presence of a house pet, home renovation in the past 2 years, area of residence per person, asthma diagnosis, family history of atopy, and short-term air pollution concentrations.

^b^ Effect expressed for a 1–interquartile range change in ambient concentration for each pollutant (PM_1_, 13.1 μg/m^3^; PM_2.5_, 10.0 μg/m^3^; PM_10_, 13.8 μg/m^3^; and NO_2_, 7.3 μg/m^3^).

## Discussion

In this large population-based study of school-aged children in northeastern China, exposure to greater air pollutant concentrations was associated with higher odds of lung function impairment. Having been breastfed and being younger were associated with less lung function impairment compared with children who were not breastfed or who were older, respectively.

To our knowledge, this is the first study to examine associations of air pollutant concentrations and breastfeeding with lung function measures among children. Our review of the literature found 3 relevant human studies that assessed breastfeeding as a modifier of the association of air pollution with respiratory responsiveness but not on lung function. A 2013 study by Dong et al^3^ found that breastfeeding moderated associations of air pollution exposure with respiratory illnesses in China. Among children who were not breastfed, greater NO_2_ concentrations were associated with higher odds ratios for cough, phlegm, current wheeze, and asthma diagnosed by a physician compared with children who were breastfed. A study by Liu et al^12^ in 2016 reported lower odds ratios of asthma associated with current environmental smoke exposure among children who were breastfed compared with children who were not breastfed. A 2003 study by Chulada et al^22^ reported on the combined associations of breastfeeding and environmental hazards (air pollution or tobacco smoke) with respiratory diseases and found significantly lower risks of asthma and wheezing among 8261 children aged 2 to 71 months who were breastfed compared with an age-matched cohort of children who were not breastfed. A 2006 study from the Czech Republic^11^ found higher risks of respiratory diseases associated with the use of coal for heating and maternal smoking among children who were not breastfed compared with children who were breastfed. Moreover, a 2008 prospective study^23^ of 1456 children observed for 10 years as members of the Isle of Wight birth cohort study reported that breastfeeding was associated with reduced incidence of asthma associated with prenatal smoking for children ages 1, 2, 4, and 10 years. In this study, we add new evidence to the literature by suggesting that breastfeeding may decrease the risk of lung function impairment from exposure to air pollution.

This study suggests that the association of breastfeeding with lung function may decrease with age.^24,25^ Other studies have also shown that the association of breastfeeding with respiratory health may diminish with age.^26,27^ A 2010 birth cohort study from Hong Kong^26^ reported that the hazard ratio for the association of breastfeeding with hospitalization for respiratory illnesses steadily increased with age. A 2012 cohort study from the Netherlands^27^ showed a higher risk of dry cough and wheezing at ages 1, 2, and 3 years for children who were not breastfed compared with children who were, but the difference diminished with age, and results were not significant among children aged 4 years.

The mechanisms for breastfeeding’s potential associations with air pollution and lung function impairment have not been established, to our knowledge. Breast milk is rich in immune factors, such as cytokines, chemokines, and maternally derived antibodies and leukocytes and may facilitate more effective pulmonary immune development and maturation than bottle feeding or early solid food consumption.^28,29,30^ Furthermore, several studies have suggested that prolonged and exclusive breastfeeding reduces respiratory infection severity and morbidity in infants, limiting virus-induced lung damage.^31,32,33^ However, the anti-infective contents of breast milk boost passive immunity only and may not provide protection after cessation of breastfeeding. The most important effect of breastfeeding may be to postpone the age at which infection and illness first occur. Studies have shown an association of respiratory illness in the first year of life with a long-term increased risk of severe respiratory illness.^34,35^ If respiratory vulnerability is established by age 3 months, then the balance of adverse and favorable factors during that critical early period could potentially affect an individual’s health throughout the life course. Another possible biological mechanism is that systemic inflammation may be caused by ambient air pollution and reduced by breast milk. Several biological pathways may be involved in oxidative stress, systemic proinflammatory responses, and the activation of pulmonary reflexes, leading to arterial remodeling of the bronchial tree.^5,36,37,38^ In addition, studies by Goldfield et al^39^ and Koenig et al^40^ suggested that infants who were breastfed may experience less ventilatory disruption than infants who were bottle-fed.^39,40^ Infant feeding requires the precise coordination of sucking, swallowing, and breathing.^41^ Prolonged suckling at the breast may convey a mechanical stimulus that results in improved ventilation mechanics associated with physical training^42^ and contribute to a larger lung capacity.^41^ Additional studies are needed to more clearly understand how breastfeeding may enhance lung volume and development.

### Strengths and Limitations

Our large sample size, which included 62 schools in 24 districts in northeastern China, is a strength of this study. Our sample size of 6740 participants represents one of the largest published studies evaluating these associations among children, to our knowledge. Furthermore, children included in the study spent a mean 11.9 minutes walking from home to school, suggesting that the children’s particulate matter exposure assessment may have represented both school and home exposure levels. For these reasons, our results may have reduced exposure uncertainty.

Although novel, the results of our study have several limitations. First, our study design was cross-sectional; thus, we could not identify a clear temporal sequence between the exposure and health outcome. However, we believe reverse causality to be improbable here, as spirometry results are unlikely to have affected the measured 4-year mean ambient air pollutant concentrations. Second, we implemented an ecological exposure assessment strategy, which was likely to have misclassified some participants living farther away from district air monitoring stations. Furthermore, air pollution measurements were more likely to reflect background levels rather than those from local sources, given mandated distances from recognized sources. As for the satellite-based data, the prediction of PM_1_, PM_2.5_, and PM_10_ from 2009 through 2012 was based on an assumption that the association of PM with its variables remained consistent over this period. However, this assumption could not be verified, as ground monitoring data were unavailable in China prior to 2013. For NO_2_, our spatial resolution of 5 km by 5 km may have been too coarse to capture high near-road exposures, which can be approximately 3-fold greater than concentrations less than 0.5 km away.^1,43^ Still, any exposure misclassification would have been similar according to lung function measurements, so the resultant bias is likely to be toward the null hypothesis. Third, we could not disentangle the relevance of the different pollutants by simultaneously including all air pollutants in the same model, considering the strong collinearity between air pollutants caused by common sources or photochemical interactions.^44^ Fourth, we did not capture data concerning indoor air pollutant concentrations,^45,46^ which have also been shown to affect children’s lung function. While adjustment for environmental tobacco smoke, home renovation, presence of a house pet, family income, and parental education likely partially mitigated confounding variables from indoor air pollutants, we cannot rule out residual confounding by these or other factors. Thus, a future study integrating a more comprehensive panel of air pollutants is necessary for a more definitive result. Fifth, we defined breastfeeding as breastfeeding for more than 3 months as reported by the mothers rather than 6 months as is usually used as the cutoff to define breastfeeding. In China, mothers have only 3 months of postpartum maternity leave, and many discontinue breastfeeding after returning to work. Mixed feeding (>1 main feeding method) may have been misclassified in some participants. Although this misclassification may have been differential according to lung function measurements, the associations changed only slightly when excluding children with mixed feeding. Sixth, our selection of schools nearest to the municipal air monitor stations may have favored clusters of children by socioeconomic status, pollution at the residence, and health conditions, which may have induced uncertain biases into our results. In addition, the statistically significant associations observed in this study need to be interpreted carefully because of the issue of multiple hypothesis testing, which needs to be taken into account. We conducted 32 statistical tests (16 tests for impaired lung function and 16 tests for continuous lung function measures) for the association of air pollutants with impaired lung function. With a 5% type I error risk for each test, the observed significant associations may be purely due to chance. However, this consideration is not likely to explain all of the significant associations.

## Conclusions

The results of this large epidemiologic study suggest that breastfeeding may be negatively associated with harmful effects of air pollution on children’s lung function in China, particularly among younger children. However, future studies are needed to confirm the results and to clarify the biological mechanisms for the associations.

## Notes

Supplement. **eMethods 1.** Description of the Study Design and Random Sampling Strategy **eMethods 2.** Description of the Anthropometry and Questionnaire Data Collection **eMethods 3.** Description of the Ground-Monitored PM_1_, PM_2.5_, PM_10_, and NO_2_ Data **eMethods 4.** Description of the PM_10_, SO_2_, NO_2_, and O_3_ Data **eMethods 5.** Description of the 2-Level Binary Logistic Regression Model **eTable 1.** Characteristics of the Study Participants and Nonparticipants **eTable 2.** Distribution of Air Pollutants, Lung Function, and Breastfeeding Prevalence Among the 7 Study Cities in China **eTable 3.** Daily Mean Air Pollutant Levels in the 24 Study Districts of the 7 Study Cities in China, 2012-2013 **eTable 4.** Crude ORs for Impaired Lung Function and 4-Year Mean Ambient Air Pollutant Concentrations Stratified by Breastfed Status **eTable 5.** Crude Estimated Absolute Change in Lung Function Test Measurements Associated With 4-Year Mean Ambient Air Pollutant Concentrations Stratified by Breastfed Status **eTable 6.** Adjusted Odds Ratios (AORs) for Impaired Lung Function and 4-Year Mean Ambient Air Pollutant Concentrations Measured by Local Air Monitoring Stations Stratified by Breastfed Status **eTable 7.** Estimated Absolute Change in Lung Function Test Measurements Associated With 4-Year Mean Ambient Air Pollutant Concentrations Measured by Local Air Monitoring Station Stratified by Breastfed Status **eTable 8.** Adjusted Odds Ratios (AORs) for Decreased Lung Function and 4-Year Mean Ambient Air Pollutant Concentrations Stratified by Breastfed Status and Excluding Children With Mixed Feeding **eTable 9.** Estimated Absolute Change in Lung Function Test Measurements Associated With 4-Year Mean Ambient Air Pollutant Concentrations Stratified by Breastfed Status and Excluding Children With Mixed Feeding **eTable 10.** Adjusted Odds Ratios (AORs) for Impaired Lung Function and 4-Year Mean Ambient Air Pollutant Concentrations Stratified by Breastfed Status and Excluding Children With Low Birth Weight or Preterm Birth **eTable 11.** Estimated Absolute Change in Lung Function Test Measurements Associated With 4-Year Mean Ambient Air Pollutant Concentrations Stratified by Breastfed Status and Excluding Children With Low Birth Weight or Preterm Birth **eTable 12.** Adjusted Odds Ratios (AORs) for Impaired Lung Function and 4-Year Mean Ambient Air Pollutant Concentrations Stratified by Breastfed Status and Excluding Children With Asthma Diagnosed by a Physician **eTable 13.** Estimated Absolute Change in Lung Function Test Measurements Associated With 4-Year Mean Ambient Air Pollutant Concentrations Stratified by Breastfed Status and Excluding Children With Asthma Diagnosed by a Physician **eTable 14.** Adjusted Odds Ratios (AORs) for Impaired Forced Vital Capacity and 4-Year Mean PM_1_ Concentrations per 1–Interquartile Range Change (13.1 μg/m^3^), Excluding Districts 1 at a Time Stratified by Breastfed Status **eFigure 1.** Associations of the Prevalence of Impaired Lung Function With Ambient Pollutants Stratified by Breastfed Status **eFigure 2.** Estimated Absolute Change in Lung Function Test Measurements Associated With 4-Year Mean Ambient Air Pollutant Concentrations Estimated Using a Spatial Statistical Model Stratified by Breastfed Status
