# Supplementary material for: Association of Breastfeeding and Air Pollution Exposure With Lung Function in Chinese Children
Source: JAMA Netw Open. 2019 May 24;2(5):e194186. doi: 10.1001/jamanetworkopen.2019.4186 (PMC6632134; doi:10.1001/jamanetworkopen.2019.4186)
Supplement: Supplement. — eMethods 1. Description of the Study Design and Random Sampling Strategy eMethods 2. Description of the Anthropometry and Questionnaire Data Collection eMethods 3. Description of the Ground-Monitored PM1, PM2.5, PM10, and NO2 Data eMethods 4. Description of the PM10, SO2, NO2, and O3 Data eMethods 5. Description of the 2-Level Binary Logistic Regression Model eTable 1. Characteristics of the Study Participants and Nonparticipants eTable 2. Distribution of Air Pollutants, Lung Function, and Breastfeeding Prevalence Among the 7 Study Cities in China eTable 3. Daily Mean Air Pollutant Levels in the 24 Study Districts of the 7 Study Cities in China, 2012-2013 eTable 4. Crude ORs for Impaired Lung Function and 4-Year Mean Ambient Air Pollutant Concentrations Stratified by Breastfed Status eTable 5. Crude Estimated Absolute Change in Lung Function Test Measurements Associated With 4-Year Mean Ambient Air Pollutant Concentrations Stratified by Breastfed Status eTable 6. Adjusted Odds Ratios (AORs) for Impaired Lung Function and 4-Year Mean Ambient Air Pollutant Concentrations Measured by Local Air Monitoring Stations Stratified by Breastfed Status eTable 7. Estimated Absolute Change in Lung Function Test Measurements Associated With 4-Year Mean Ambient Air Pollutant Concentrations Measured by Local Air Monitoring Station Stratified by Breastfed Status eTable 8. Adjusted Odds Ratios (AORs) for Decreased Lung Function and 4-Year Mean Ambient Air Pollutant Concentrations Stratified by Breastfed Status and Excluding Children With Mixed Feeding eTable 9. Estimated Absolute Change in Lung Function Test Measurements Associated With 4-Year Mean Ambient Air Pollutant Concentrations Stratified by Breastfed Status and Excluding Children With Mixed Feeding eTable 10. Adjusted Odds Ratios (AORs) for Impaired Lung Function and 4-Year Mean Ambient Air Pollutant Concentrations Stratified by Breastfed Status and Excluding Children With Low Birth Weight or Preterm Birth eTable 11. Estimated [file jamanetwopen-2-e194186-s001.pdf]

# Supplementary Online Content

Zhang C, Guo Y, Xiao X, et al. Association of breastfeeding and air pollution exposure with lung function in Chinese children. *JAMA Netw Open*. 2019;2(5):e194186. doi:10.1001/jamanetworkopen.2019.4186

**eMethods 1.** Description of the Study Design and Random Sampling Strategy

**eMethods 2.** Description of the Anthropometry and Questionnaire Data Collection

**eMethods 3.** Description of the Ground-Monitored $PM_1$, $PM_{2.5}$, $PM_{10}$, and $NO_2$ Data

**eMethods 4.** Description of the $PM_{10}$, $SO_2$, $NO_2$, and $O_3$ Data

**eMethods 5.** Description of the 2-Level Binary Logistic Regression Model

**eTable 1.** Characteristics of the Study Participants and Nonparticipants

**eTable 2.** Distribution of Air Pollutants, Lung Function, and Breastfeeding Prevalence Among the 7 Study Cities in China

**eTable 3.** Daily Mean Air Pollutant Levels in the 24 Study Districts of the 7 Study Cities in China, 2012-2013

**eTable 4.** Crude Odd Ratios (ORs) for Impaired Lung Function and 4-Year Mean Ambient Air Pollutant Concentrations Stratified by Breastfed Status

**eTable 5.** Crude Estimated Absolute Change in Lung Function Test Measurements Associated With 4-Year Mean Ambient Air Pollutant Concentrations Stratified by Breastfed Status

**eTable 6.** Adjusted Odds Ratios (AORs) for Impaired Lung Function and 4-Year Mean Ambient Air Pollutant Concentrations Measured by Local Air Monitoring Stations Stratified by Breastfed Status

**eTable 7.** Estimated Absolute Change in Lung Function Test Measurements Associated With 4-Year Mean Ambient Air Pollutant Concentrations Measured by Local Air Monitoring Station Stratified by Breastfed Status

**eTable 8.** Adjusted Odds Ratios (AORs) for Decreased Lung Function and 4-Year Mean Ambient Air Pollutant Concentrations Stratified by Breastfed Status and Excluding Children With Mixed Feeding

**eTable 9.** Estimated Absolute Change in Lung Function Test Measurements Associated With 4-Year Mean Ambient Air Pollutant Concentrations Stratified by

© 2019 Zhang C et al. *JAMA Network Open*.

Breastfed Status and Excluding Children With Mixed Feeding

**eTable 10.** Adjusted Odds Ratios (AORs) for Impaired Lung Function and 4-Year Mean Ambient Air Pollutant Concentrations Stratified by Breastfed Status and Excluding Children With Low Birth Weight or Preterm Birth

**eTable 11.** Estimated Absolute Change in Lung Function Test Measurements Associated With 4-Year Mean Ambient Air Pollutant Concentrations Stratified by Breastfed Status and Excluding Children With Low Birth Weight or Preterm Birth

**eTable 12.** Adjusted Odds Ratios (AORs) for Impaired Lung Function and 4-Year Mean Ambient Air Pollutant Concentrations Stratified by Breastfed Status and Excluding Children With Asthma Diagnoses by a Physician

**eTable 13.** Estimated Absolute Change in Lung Function Test Measurements Associated With 4-Year Mean Ambient Air Pollutant Concentrations Stratified by Breastfed Status and Excluding Children With Asthma Diagnoses by a Physician

**eTable 14.** Adjusted Odds Ratios (AORs) for Impaired Forced Vital Capacity and 4-Year Mean $PM_1$ Concentrations per 1–Interquartile Range Change (13.1 μg/$m^3$), Excluding Districts 1 at a Time Stratified by Breastfed Status

**eFigure 1.** Associations of the Prevalence of Impaired Lung Function With Ambient Pollutants Stratified by Breastfed Status.

**eFigure 2.** Estimated Absolute Change in Lung Function Test Measurements Associated With 4-Year Mean Ambient Air Pollutant Concentrations Estimated Using a Spatial Statistical Model Stratified by Breastfed Status

This supplementary material has been provided by the authors to give readers additional information about their work.

© 2019 Zhang C et al. *JAMA Network Open*.

## **eMethods 1.** Description of the Study Design and Random Sampling Strategy

The Seven Northeastern Cities (SNEC) study is a cross-sectional study of children's health outcomes based on exposure to ambient air pollutants. This region encompasses more than 20million people residing in 14 cities in Liaoning province in Northeastern China. To maximize the inter- and intra-city gradients of the pollutants of interest and also to minimize the correlation between district-specific ambient pollutants, in April 2012 the seven cities of Shenyang, Dalian, Anshan, Fushun, Benxi, Liaoyang, and Dandong in Liaoning province were selected as study sites, based on air pollution measurements taken between 2009 and 2011 (Map 1). In each of the seven cities, we selected all urban districts for the study. There are five districts in Shenyang, four districts in Dalian and Fushun, three districts in Anshan, Benxi, and Dandong, and two districts in Liaoyang, respectively.

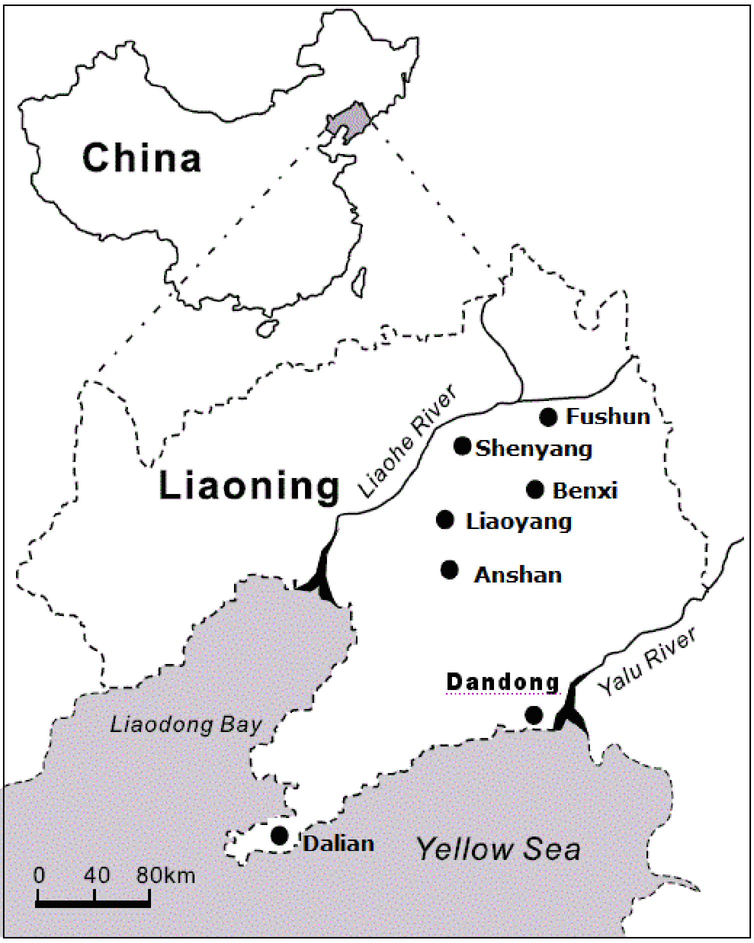


**Map 1.** Locations of the study cities in Liaoning province, northeast of China

In each of the 24 study districts, there was only one available municipal air monitoring station. To generate a representative sample, we randomly selected one or two elementary schools and one or two middle schools, located within 2 km of a municipal monitoring station. If the number of students in the first selected school was below 500, we included a second school. The resulting 62 schools were included. Within each school, we randomly selected one or two classrooms depending on the class size from each grade level to enroll study participants.

The Map 2 below shows an area including up to a 2 km radius around one of our monitoring stations,

© 2019 Zhang C et al. *JAMA Network Open*.

as indicated by a small, brown circle in the center of the map. As shown in the map, 9 elementary schools (marked 1-9) were within a 2 km radius of that particular station. We randomly selected one or two elementary schools from those 9 by using a random number table.

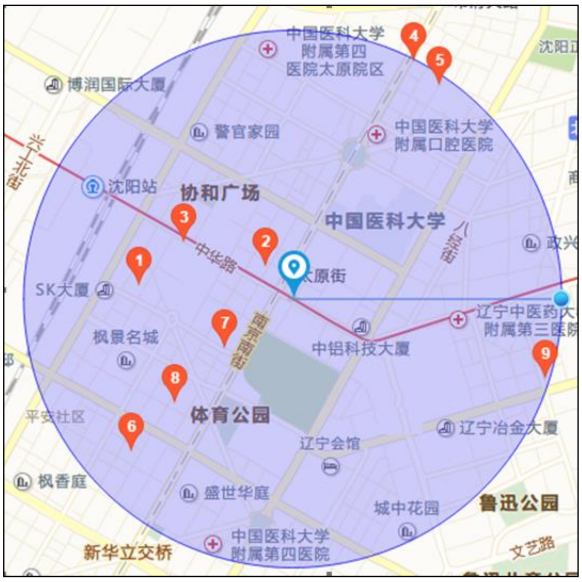


**Map 2.** The area including a 2 km radius around a monitoring station, as indicated by a small, blue circle in the center of the map. Elementary schools, marked 1-9, fell within this radius. We randomly selected school 1 and 9.

In China, each district of a city has an administrative code. We selected the last two non-zero valid digits of the administrative code as the number of the district and randomly selected two schools based on a random number table. For example, the administrative code of the Heping district in Shenyang is "210102000000." We identified "02" as the number of the Heping district. According to a random number table, as shown below, the random numbers "1" and "9" were selected in this survey. So, schools with label 1 and 9 in the figure were included. These randomization procedures ensure that the schools selected are representative of the region.

**随机数字表 Random Number Table**

| 编号 | 1~10 | 11~20 | 21~30 | 31~40 | 41~50 |
|---|---|---|---|---|---|
| 1 | 22 17 68 65 81 | 68 95 23 92 35 | 87 02 22 57 51 | 61 09 43 95 06 | 58 24 82 03 47 |
| 2 | 19 36 27 59 46 | 13 79 93 37 55 | 39 77 32 77 09 | 85 52 05 30 62 | 47 83 51 62 74 |
| 3 | 16 77 23 02 77 | 09 61 84 25 21 | 28 06 24 25 93 | 16 71 13 59 78 | 23 05 47 47 25 |
| 4 | 78 43 76 71 61 | 20 44 90 32 64 | 97 67 63 99 61 | 46 38 03 93 22 | 69 81 21 99 21 |
| 5 | 03 28 28 26 08 | 73 37 32 04 05 | 69 30 16 09 05 | 88 69 58 28 99 | 35 07 44 75 47 |

In China, primary schools admit students restrictively basing the criteria on geographical boundaries, and the policy forbids selecting trans-regionally schools for children. Therefore, the monitoring station, which was the only station in the selected child's school district, was the nearest station to the child's home. Furthermore, in the present study, the average time walking from home to school was only 11.9 minutes for the study school children, indicating the children's PM exposure assessment may represent both school and home exposure levels. For these reasons, our results may have reduced exposure uncertainty.

© 2019 Zhang C et al. *JAMA Network Open*.

## **eMethods 2.** Description of the Anthropometry and Questionnaire Data Collection

The study questionnaire included queries about demographics, lifestyle factors in the home, family medical history, health-related behaviors in the home, and household environmental factors such as home coal use, house pet, home renovation in the past 2 years, area of residence per person. The trainees and investigators in the study were required to complete a training program on a standardized protocol for the physical examination. Following training, each trainee had to take a mandatory qualifying examination to ensure proper administration of the study questionnaire and adherence to the standardized protocol, and those who passed were awarded a Physical Observer Certificate. Each of the participating children completed a physical examination in school from April 2012 to May 2013. Trained nurses followed the standardized World Health Organization protocol for measuring children's height (at 0.1cm) and weight (at 0.1kg) and used them to calculate the body mass index (BMI) in kg/$m^2$. We dichotomized parent's education as having a high school education or not, and categorized annual family income as: < 5000 Chinese Yuan (RMB), 5000-9,999 RMB, 10,000-29,999 RMB, 30,000-99,999 RMB, or >100,000 RMB. Preterm birth was defined as a gestational age < 37 weeks at delivery. Low birth weight was characterized as a birth weight < 2500 g. Children's passive tobacco smoke exposure was defined as cohabitation with a daily cigarette smoker, and home coal use was assessed as household use for cooking or space heating. Area of residence per person ($m^2$) was calculated by dividing the housing area by the number of household residents. We also queried the 'ever' presence of a house pet (i.e., dog, cat, bird, farm animals, and others). We defined family history of atopy as a clinical diagnosis of allergies (e.g., allergic dermatitis): hay fever, asthma, or bronchial asthma in a biologic parent or grandparent. Doctor-diagnosed asthma was defined as an affirmative answer to the question "Has a doctor ever diagnosed asthma in this child?" Current asthma among previously asthma-diagnosed children was defined by an affirmative answer to the question ''Has this child had an asthma attack in the last two years?'' or an affirmative answer to the question ''Does this child take medicine or treatment for asthma or asthmatic bronchitis?'' We determined home renovation as a positive response to the question "Have you made any renovations in your home within the past 2 years?"

We defined breastfeeding as having been mainly breastfed for more than three months. In present study, new mothers had a 3-month pregnancy leave after delivery. After 3 months, most of the new mothers had to return to work and many of them discontinued breastfeeding at that time. Thus, we designed the question to assess only 3-month breastfeeding information. Mainly breastfed means that the child was fed mainly by breast milk, and occasionally fed by others. The early diet of children not breastfed includes animal milk, juice, and soups made from egg, rice, chicken, pork, beef, fish, or vegetables. During investigation, we use the following two questions to collect the information of breastfeeding status: the first questions, "Which of the following breastfeeding methods is used within 3 months after birth (multiple options): A. Breast milk; B. animal milk (mainly from cow); C, others (including: juice, and soups made from egg, rice, chicken, pork, beef, fish, or vegetables)". The second question, "if you select more than one option, sort by importance". In present study, a total of 4,751 (70.5%) parents reported breastfeeding including 302 mixed feeding (select [A and B], or [A and C]).

© 2019 Zhang C et al. *JAMA Network Open*.

## **eMethods 3.** Description of the Ground-Monitored $PM_1$, $PM_{2.5}$, $PM_{10}$, and $NO_2$ Data

Ground-monitored airborne particulate matter with a diameter of 1 μm or less ($PM_1$), airborne particulate matter with a diameter of 2.5 μm or less ($PM_{2.5}$) and airborne particulate matter with a diameter of 10 μm or less ($PM_{10}$) were obtained from the China Atmosphere Watch Network (CAWNET) of the China Meteorological Administration (CMA). The network consisted of 96 stations across mainland China. Concentrations of $PM_1$, $PM_{2.5}$ and $PM_{10}$ at all stations were measured with GRIMM 180 Environmental dust monitors (Model 1.108, Grimm Aerosol Technik GmbH, Ainring, Germany). Daily concentration of $NO_2$ was estimated with satellite-derived OMI data (Daily Level-3 Nitrogen Dioxide Product) and other predictors. Two quality-control procedures were applied to all PM measurements: a "limit check" and "climatological check". For the limit check, we evaluated each valid PM measurement to determine whether it fell within its possible limits, otherwise, they were removed. In the climatological check, the median and standard deviation (SD) of hourly PM measurements were calculated at each PM observational site. Any PM values lying outside of more than three SDs from the median PM have been removed. Daily $PM_1$, $PM_{2.5}$, $PM_{10}$, and $NO_2$ concentrations were estimated by using a spatial statistical model with a machine learning method matched to the children's geocoded home addresses. Briefly, each participant's home address was geocoded as a geographical longitude and latitude, and superimposed over the predicted daily $PM_1$, $PM_{2.5}$, $PM_{10}$, and $NO_2$ concentrations' grids, and then the exposure parameters were calculated by averaging the daily concentrations for $PM_1$, $PM_{2.5}$, $PM_{10}$, and $NO_2$ over the four-year period of 2009–2012.

This method is user-friendly, as there is no need to define the complex relationships between predictors (e.g., linear or nonlinear relationships and interactions). Also, the variable importance measures provided by random forests help the user to identify important variables and noise variables. The final model is shown as following:

$$PM_{ij} = AOD_{ij} + TEMP_{ij} + RH_{ij} + BP_{ij} + WS_{ij} + NDVI + Urban\_cover + doy + \log(elev)$$

$$NO_{2ij} = OMI_{ij} + TEMP_{ij} + BP_{ij} + RH_{ij} + WS_{ij} + NDVI_{ij} + Urban\_cover_{ij} + doy_i + log(elev_j)$$

where $PM_{2.5ij}$ is the $PM_{2.5}$ or $PM_{10}$ concentration on day *i* at station *j*; $NO_{2ij}$ is the $NO_2$ concentration on day *i* at station *j*; $AOD_{ij}$ is the combined AOD; $OMI_{ij}$ is the satellite-derived OMI value; *TEMP*, *RH*, *BP*, and *WS* are mean temperature, relative humidity, barometric pressure, and wind speed on day i, respectively; *NDVI* is the monthly average NDVI value; *Urban_cover* is the percentage of urban cover with a buffer radius of 10 km; *doy* is day of the year; and *log(elev)* is the log transformed elevation. To evaluate the predictive ability of the final model, a 10-fold cross-validation (CV) was performed.

The results of a 10-fold cross-validation showed $R^2$ values for daily and annual predictions were 55% and 75% for $PM_1$, 83% and 86% for $PM_{2.5}$, 78% and 81% for $PM_{10}$, and 64% and 72% for $NO_2$, respectively. The Root Mean Squared Error (RMSE) values for daily and annual predictions were 20.5 μg/m$^3$ and 8.8 μg/m$^3$ for $PM_1$, 18.1 μg/m$^3$ and 6.9 μg/m$^3$ for $PM_{2.5}$, 31.5 μg/m$^3$ and 14.4 μg/m$^3$ for $PM_{10}$, and 12.4μg/m$^3$ and 6.5 μg/m$^3$ for $NO_2$, respectively.

© 2019 Zhang C et al. *JAMA Network Open*.

## **eMethods 4.** Description of the $PM_{10}$, $SO_2$, $NO_2$, and $O_3$ Data

The operation of the monitoring stations has strictly followed the quality assurance/quality control (QA/QC) procedure set by the State Environmental Protection Administration of China (SEPAC,1992). The environmental monitoring centers in each of the three cities conducted regularly performance audits and precision checks on the air-monitoring equipment. Quarterly performance audits are conducted to assess data accuracy on airborne particulate matter with a diameter of 10 μm or less ($PM_{10}$), sulfur dioxide ($SO_2$), nitrogen dioxide ($NO_2$), and ozone ($O_3$) monitoring systems.

1) The calculation method

   The calculation method is performed according to Chinese National standards (GB8170-87). The unit of monitored pollutants is $mg/m^3$ accurate to the third decimal. The units can also be expressed as $\mu g/m^3$, depending on the pollutant's concentration. For concentrations that were too low to be measured, half of the lowest checking limit of the equipment will be used as the measured value.

2) Outliers

   When the measured concentration is too low (e.g. background value), a negative value can be obtained because of the zero drift of the monitor. There is no physical meaning to this value. This negative value can be regarded as a value of "unable to measure."

   For the monitoring station with an automatic calibration system, if equipment zero drift/span drift exceeds the control range during the period of zero/span calibration, the data from the time it becomes out of control until the equipment is recovered should be regarded as invalid data. The data cannot be used statistically.

   The data during the period of zero calibration/span calibration should be regarded as invalid data. It cannot be used statistically, but a flag should be made on these data and the records stored as evidence.

   When values are missing because of a loss of power, any data received by the central control station during the period of the loss of power should be regarded as invalid data. The period of loss of power should be counted at the start of power outage until complete warm-up of equipment. The data cannot be used statistically.

   Because pollutant concentrations change over time and change slowly, there should be no swift change in pollutant concentration in the results of normal monitoring. Either a swift change or no change indicates that there is an equipment problem. The problem should be identified, and the data between the start of problem to recovery should be regarded as outliers. These data cannot be used statistically.

3) Statistics of monitoring data

   One time value

© 2019 Zhang C et al. *JAMA Network Open*.

The central control station uses an average of 15 minutes of pollutant concentrations measured at the branch station as a one-time value. The central control modifies this value and judge whether this value is an outlier using the report software.

One hour mean value

At least 75% of the one-time values should be used to calculate the one-hour average mean value. One-hour average mean value is calculated by averaging all the valid one-time values within one hour.

Daily average mean

For $PM_{10}$ at least 12 valid hourly mean values are needed to calculate the daily mean value (using the calendar as the valid time frame), using all available valid hourly mean values. For $SO_2$ and $NO_2$ at least 18 hourly mean values everyday are needed to calculate valid daily mean value (using the calendar as the valid time frame). For $O_3$, at least six hourly concentrations of $O_3$ per day are needed for calculating the 8-hour average concentration of $O_3$. All of the valid hourly mean values are used to calculate the daily mean. (National Environmental Air Quality Standard GB3095-2012)

Monthly mean values are the arithmetic means of all valid daily mean values within the month. Seasonal mean values are the arithmetic means of all valid daily mean values within the season. Yearly mean values are the arithmetic means of all valid daily mean values within the year. District daily mean values are calculated using the monthly mean value, the seasonal mean value, and the yearly mean value from the available stations in the district.

© 2019 Zhang C et al. *JAMA Network Open*.

## **eMethods 5.** Description of the 2-Level Binary Logistic Regression Model

We assessed normality and described distributions for continuous variables as the mean ± standard deviation (SD), and categorical variables as n (%), comparing breastfed to non-breastfed children by Student's *t*-test or $\chi^2$-test as appropriate. To investigate the relationship between the pulmonary function tests (PFT) and ambient air pollution, we considered a two-level logistic regression model in which children were the first-level units and the districts were the second-level units. At the child level, we predicted the logit of the prevalence rate for a given impaired lung function outcome by breastfeeding (BF) and k covariates (X1 ….Xk) as follows:

$$\text{logit}\ [P(\text{symptom}_{ij})] = \alpha_j + \lambda_j BF_{ij} + \beta_1 X1_{ij} + \ldots + \beta_k X1_{ij} + e_{ij} \qquad (1)$$

where the subscript j is for districts (j=1,…, 25), the subscript i is for children (i=1,..$n_j$), $\alpha_j$ are the intercepts at the district level, λj are the regression coefficients for breastfeeding, $\beta_1 \ldots \beta_k$ are the regression coefficients of covariates, and $e_{ij}$ are the random errors, assumed to have mean of zero and constant variance. The $\alpha_j$ and $\lambda_j$ are random coefficients as they are assumed to vary across districts. In general, a district with a high $\alpha_j$ is predicted to have higher prevalence rates than a district with a low $\alpha_j$. Similarly, differences in $\lambda_j$ indicate that the relationship between breastfeeding and prevalence rates is not the same in all districts. In districts with a high (low) $\lambda_j$, breastfeeding has a large (small) effect on prevalence rates (i.e., the difference between breastfed children and non-breastfed children is relatively large (small)). At the district level, we regressed the district-specific intercepts $\alpha_j$ and coefficients $\lambda_j$ on the district-specific pollutant level ($Z_j$) to explain the variations of $\alpha_j$ and $\lambda_j$, as follows:

$$\alpha j = \alpha + \gamma 1 Z_j + u1_j \qquad (2)$$

$$\lambda_j = \lambda + \gamma 2 Z_j + u2_j \qquad (3)$$

Equation (2) predicts the prevalence rates in a district by $Z_j$. If γ1 is positive, then adjusting for covariates, the prevalence rates are higher in districts with a higher pollutant level. Conversely, if $\gamma_1$ is negative, then adjusting for covariates, the prevalence rates are lower in districts with a higher pollutant level. Equation (3) states that, adjusting for covariates, the relationship between prevalence rates and breastfeeding in a district depends on the district's pollutant level $Z_j$. If γ2 is positive, then adjusting for covariates, the breastfeeding effect on prevalence rates is larger with a higher pollutant level. Conversely, if γ2 is negative, then adjusting for covariates, the breastfeeding effect on prevalence rates is smaller with a higher pollutant level. The -terms $u1_j$ and $u2_j$ are random errors at the district level, assumed to be independent and have mean of zero and constant variance. These random errors characterize the between-district variation and are assumed to be independent from $e_{ij}$ at the child level. Note that α, λ, β1,…, βk, γ1, and γ2 are fixed effects and so do not vary across districts (they therefore have no subscript j to indicate district). The above models can be written as a single regression equation by substituting equations (2) and (3) into equation (1):

$$\text{logit}[P(\text{symptomj})]=(\alpha+\gamma 1\ Zj+\lambda\ BFij+\beta 1X1ij+ \ldots+\beta kXkij+\gamma 2Zj\ BFij)$$
$$+(u2jBFij+u1j+ eij) \qquad (4)$$

The terms in the first and second parentheses in equation (4) are often called the fixed (or deterministic) and random (or stochastic) parts of the model, respectively. The product term ZjBFij is a cross-level interaction between the child-level variable BFij and the district-level variable Zj. The random error u2j BFij is different for different children, a situation that in ordinary multiple regression analysis is called heteroscedasticity. To evaluate the robustness of our estimates, we conducted a

© 2019 Zhang C et al. *JAMA Network Open*.

number of sensitivity analyses, including stratifying by child's age, excluding children with mixed feeding, excluding children with low birth weight or preterm birth, excluding children with doctor-diagnosed asthma, and randomly excluding one district. Analyses were also adjusted for a potential confounders selected *a priori* based on literature evidence for associations with air pollution exposure and respiratory function. These factors included age, sex, height, birth weight, preterm birth, parental education, family income per year, exercise per week, passive smoke exposure, home coal use, house pet, home renovation in the past 2 years, area of residence per person, doctor-diagnosed asthma, family history of atopy, and short-term air pollution concentrations. Short-term air pollution concentrations referred to the daily mean pollutant levels from routine air monitoring data during lung function measurement in the children. If the estimated regression effects for pollutants changed by at least 10% upon inclusion in the base model, the covariate was retained in the final model as a confounding variable. All analyses were conducted using the GLIMMIX procedure in SAS 9.4 (SAS Institute, Cary, NC USA). All statistical tests were two-tailed, and *p*-values less than 0.05 were considered statistically significant except for the interaction term where statistical significance was asserted at *p*-values less than 0.1.

© 2019 Zhang C et al. *JAMA Network Open*.

**eTable 1.** Characteristics of the Study Participants and Nonparticipants

| Characteristics | Participants (n=6,740) | Non-participants (n=279) |
|---|---|---|
| Age (years); mean (SD) | 11.6 (2.1) | 11.7 (2.3) |
| Height (cm); mean (SD) | 153.9 (12.6) | 153.9 (14.0) |
| Weight (kg); mean (SD) | 48.4 (15.6) | 47.9 (17.7) |
| Body mass index (kg/m$^2$); mean (SD) | 20.0 (4.7) | 20.5 (7.8) |
| Birth weight (kg); mean (SD) | 3.4 (0.5) | 3.4 (0.5) |
| Area of residence per person (m$^2$); mean (SD) | 22.7 (9.8) | 22.7 (9.8) |
| Exercise per week (hours); mean (SD) | 7.6 (7.7) | 7.9 (8.9) |
| Male | 3,382 (50.2) | 146 (52.3) |
| Preterm birth | 338 (5.0) | 14 (5.0) |
| Parental education ≥ high school* | 4,211 (62.5) | 140 (50.2) |
| Family income per year | | |
| < 5,000 RMB | 758 (11.2) | 41 (14.7) |
| 5,000-9,999 RMB | 876 (13.0) | 43 (15.4) |
| 10,000-29,999 RMB | 2,394 (35.5) | 103 (36.9) |
| 30,000-99,999 RMB | 2,437 (36.2) | 85 (30.5) |
| > 100,000 RMB | 275 (4.1) | 7 (2.5) |
| Passive smoke exposure | | |
| Father | 2,122 (31.5) | 94 (33.7) |
| Mother | 608 (9.0) | 21 (7.5) |
| Other | 551 (8.2) | 16 (5.7) |
| Anyone | 3,281 (48.7) | 131 (46.9) |
| Mother smoking during pregnancy | 54 (0.80) | |
| Home coal use | 676 (10.0) | 38 (13.6) |
| Pet kept in home | 1,435 (21.3) | 65 (23.3) |
| Home renovation in past 2 years | 2,416 (35.9) | 114 (40.9) |
| Family history of atopy | 1,390 (20.6) | 52 (18.6) |
| Doctor-diagnosed asthma | 460 (6.8) | 17 (6.1) |
| Current asthma | 297 (4.4) | 12 (4.3) |

Abbreviations: RMB, Chinese Yuan; SD, standard deviation.

Values are *n* (%) except where indicated.

*Significant difference exists between breastfed and non-breastfed children as tested by $\chi^2$ test for categorical variables and Student's *t*-test for continuous variables, $p$ value <0.05.

© 2019 Zhang C et al. *JAMA Network Open*.

**eTable 2.** Distribution of Air Pollutants, Lung Function, and Breastfeeding Prevalence Among the 7 Study Cities in China

| | | Shenyang | Dalian (n=510) | Fushun (n=269 | Anshan (n=649) | Benxi (n=674) | Dandong | Liaoyang |
|---|---|---|---|---|---|---|---|---|
| **Air pollutants estimated using a spatial statistical model** | | | | | | | | |
| $PM_1$ | Means (SD) | 45.16 | 42.94 | 48.02 | 51.76 | 45.23 | 44.82 | 43.99 |
| | (Min~Max) | (38.35- | (39.80- | (38.35- | (38.35- | (38.94- | (38.15- | (38.63- |
| $PM_{2.5}$ | Means (SD) | 52.63 | 50.24 | 54.93 | 59.54 | 51.65 | 51.38 | 51.84 |
| | (Min~Max) | (46.76- | (46.83- | (46.61- | (46.42- | (46.42- | (46.04- | (47.85- |
| $PM_{10}$ | Means (SD) | 94.13 | 90.71 | 97.47 | 104.09 | 90.34 | 91.56 | 93.50 |
| | (Min~Max) | (83.17- | (82.91- | (83.17- | (75.90- | (75.90- | (82.64- | (87.29- |
| $NO_2$ | Means (SD) | 32.85 | 32.49 | 34.91 | 36.27 | 30.67 | 31.73 | 32.41 |
| | (Min~Max) | (27.77- | (27.64- | (27.77- | (20.57- | (20.57- | (27.72- | (29.68- |
| **Air pollutants measured by local air monitoring station** | | | | | | | | |
| $PM_{10}$ | Means (SD) | 107.43 | 81.89 | 81.48 | 86.12 | 94.61 | 87.81 | 70.82 |
| | (Min~Max) | (101.00- | (56.00- | (67.75- | (76.50- | (79.50- | (50.00- | (68.75- |
| $SO_2$ | Means (SD) | 68.06 | 44.26 | 50.07 | 49.98 | 51.34 | 40.24 | 30.85 |
| | (Min~Max) | (60.00- | (43.25- | (37.75- | (30.00- | (35.50- | (14.75- | (25.25- |
| $NO_2$ | Means (SD) | 35.56 | 46.45 | 36.68 | 26.31 | 41.54 | 41.66 | 25.45 |
| | (Min~Max) | (31.75- | (31.75- | (28.00- | (10.50- | (32.25- | (34.75- | (23.50- |
| $O_3$ | Means (SD) | 70.64 | 48.35 | 32.19 | 49.42 | 330.94 | 237.98 | 32.66 |
| | (Min~Max) | (50.25- | (15.00- | (24.00- | (45.50- | (30.31- | (27.00- | (29.50- |
| **Spirometric parameters** | | | | | | | | |
| FVC (L); means | | 2.62 | 2.74 | 2.65 | 2.53 | 2.40 | 2.75 | 2.58 |
| $FEV_1$ (L); means | | 2.44 | 2.58 | 2.45 | 2.40 | 2.26 | 2.67 | 2.51 |
| PEF (L/s); means | | 4.65 | 5.09 | 4.78 | 4.69 | 4.56 | 4.98 | 4.79 |
| MMEF (L/s); | | 3.29 | 3.61 | 3.23 | 3.36 | 3.14 | 3.67 | 3.75 |
| **Lung function status** | | | | | | | | |
| FVC, <85% of | | 13.35 | 9.61 | 9.98 | 11.40 | 13.20 | 13.16 | 7.49 |
| $FEV_1$, <85% of | | 10.82 | 7.65 | 8.46 | 6.93 | 9.50 | 8.25 | 4.87 |
| PEF, <75% of | | 9.47 | 6.27 | 5.86 | 5.55 | 5.64 | 9.57 | 3.37 |
| MMEF, <75% of | | 11.18 | 7.45 | 10.69 | 6.93 | 8.90 | 8.37 | 3.37 |
| **Prevalence of** | | 67.36 | 73.33 | 69.39 | 70.72 | 68.84 | 76.20 | 74.91 |

© 2019 Zhang C et al. *JAMA Network Open*.

**eTable 3.** Daily Mean Air Pollutant Levels in the 24 Study Districts of the 7 Study Cities in China, 2012-2013

| Pollutants | Mean | Median | Max | Min | Interquartile range |
|---|---|---|---|---|---|
| $PM_{10}$ (μg/$m^3$) | 108.8 | 108.6 | 189 | 45.2 | 47.4 |
| $SO_2$ (μg/$m^3$) | 30.2 | 24.6 | 80.4 | 4.4 | 22.8 |
| $NO_2$ (μg/$m^3$) | 26.9 | 25.5 | 64 | 3.6 | 18.6 |
| $O_3$ (μg/$m^3$) | 69 | 69.4 | 132.4 | 19 | 51.4 |

Abbreviations: $O_3$, ozone; $NO_2$, nitrogen dioxides; $PM_{10}$, airborne particulates with aerodynamic diameter ≤10 μm; $SO_2$, sulfur dioxide.

© 2019 Zhang C et al. *JAMA Network Open*.

**eTable 4.** Crude ORs for Impaired Lung Function and 4-Year Mean Ambient Air Pollutant Concentrations Stratified by Breastfed Status

| Pollutant | Non-breastfed | Breastfed | Interaction |
|---|---|---|---|
| | OR (95%CI)[a] | OR (95%CI)[a] | *P* Value |
| FVC < 85% of predicted value | | | |
| Air Pollutants estimated using a spatial statistical model | | | |
| $PM_1$, μg/m$^3$ | 3.04 (2.21-4.16) | 1.25 (1.00-1.55) | <0.001 |
| $PM_{2.5}$, μg/m$^3$ | 2.73 (2.09-3.57) | 1.29 (1.06-1.56) | <0.001 |
| $PM_{10}$, μg/m$^3$ | 2.23 (1.78-2.80) | 1.48 (1.25-1.77) | 0.004 |
| $NO_2$, μg/m$^3$ | 2.11 (1.65-2.70) | 1.68 (1.38-2.06) | 0.142 |
| Air Pollutants measured by local air monitoring station | | | |
| $PM_{10}$, μg/m$^3$ | 2.15 (1.69-2.75) | 1.31 (1.09-1.56) | 0.001 |
| $SO_2$, μg/m$^3$ | 1.55 (1.13-2.12) | 1.18 (0.92-1.53) | 0.120 |
| $NO_2$, μg/m$^3$ | 1.30 (1.01-1.66) | 1.08 (0.88-1.34) | 0.178 |
| $O_3$, μg/m$^3$ | 1.08 (1.03-1.13) | 1.03 (0.99-1.08) | 0.099 |
| FEV1 < 85% of predicted value | | | |
| Air Pollutants estimated using a spatial statistical model | | | |
| $PM_1$, μg/m$^3$ | 3.19 (2.23-4.57) | 1.47 (1.14-1.91) | 0.001 |
| $PM_{2.5}$, μg/m$^3$ | 3.21 (2.37-4.36) | 1.51 (1.21-1.88) | <0.001 |
| $PM_{10}$, μg/m$^3$ | 2.77 (2.15-3.58) | 1.73 (1.42-2.11) | 0.003 |
| $NO_2$, μg/m$^3$ | 2.81 (2.12-3.72) | 2.08 (1.65-2.61) | 0.085 |
| Air Pollutants measured by local air monitoring station | | | |
| $PM_{10}$, μg/m$^3$ | 2.08 (1.52-2.83) | 1.59 (1.23-2.06) | 0.183 |
| $SO_2$, μg/m$^3$ | 1.50 (1.08-2.10) | 1.25 (0.86-1.81) | 0.375 |
| $NO_2$, μg/m$^3$ | 1.27 (0.94-1.72) | 1.23 (0.94-1.61) | 0.841 |
| $O_3$, μg/m$^3$ | 1.07 (1.00-1.14) | 1.03 (0.96-1.09) | 0.254 |
| PEF < 75% of predicted value | | | |
| Air Pollutants estimated using a spatial statistical model | | | |
| $PM_1$, μg/m$^3$ | 1.64 (1.17-2.30) | 1.26 (0.97-1.64) | 0.204 |
| $PM_{2.5}$, μg/m$^3$ | 1.48 (1.12-1.96) | 1.21 (0.96-1.52) | 0.246 |
| $PM_{10}$, μg/m$^3$ | 1.37 (1.08-1.75) | 1.21 (0.98-1.50) | 0.404 |
| $NO_2$, μg/m$^3$ | 1.42 (1.09-1.86) | 1.32 (1.05-1.67) | 0.669 |
| Air Pollutants measured by local air monitoring station | | | |
| $PM_{10}$, μg/m$^3$ | 1.82 (1.33-2.49) | 1.67 (1.29-2.17) | 0.598 |
| $SO_2$, μg/m$^3$ | 1.47 (1.05-2.06) | 1.24 (0.84-1.85) | 0.369 |
| $NO_2$, μg/m$^3$ | 1.64 (1.20-2.23) | 1.27 (0.98-1.65) | 0.082 |
| $O_3$, μg/m$^3$ | 1.06 (1.00-1.13) | 1.05 (0.98-1.11) | 0.551 |
| MMEF < 75% of predicted value | | | |
| Air Pollutants estimated using a spatial statistical model | | | |
| $PM_1$, μg/m$^3$ | 1.50 (1.09-2.04) | 1.23 (0.98-1.55) | 0.314 |
| $PM_{2.5}$, μg/m$^3$ | 1.41 (1.09-1.83) | 1.24 (1.02-1.51) | 0.427 |
| $PM_{10}$, μg/m$^3$ | 1.36 (1.09-1.70) | 1.30 (1.08-1.55) | 0.729 |
| $NO_2$, μg/m$^3$ | 1.36 (1.06-1.74) | 1.39 (1.13-1.70) | 0.896 |
| Air Pollutants measured by local air monitoring station | | | |
| $PM_{10}$, μg/m$^3$ | 1.75 (1.30-2.35) | 1.67 (1.31-2.13) | 0.782 |
| $SO_2$, μg/m$^3$ | 1.71 (1.28-2.28) | 1.19 (0.84-1.68) | 0.029 |
| $NO_2$, μg/m$^3$ | 1.44 (1.09-1.90) | 1.31 (1.04-1.66) | 0.482 |
| $O_3$, μg/m$^3$ | 1.05 (0.98-1.11) | 1.04 (0.99-1.10) | 0.934 |

© 2019 Zhang C et al. *JAMA Network Open*.

Abbreviations: CI, confidence interval; $FEV_1$, forced expiratory volume in 1 second; FVC, forced vital capacity; MMEF, maximum mid expiratory flow rate; $O_3$, ozone; OR, odds ratio; $NO_2$, nitrogen dioxide; PEF, peak expiratory flow rate; $PM_1$, airborne particulates with aerodynamic diameter ≤1 μm; $PM_{2.5}$, ≤2.5 μm, $PM_{10}$, ≤10 μm; $SO_2$, sulfur dioxide.
[a]Effect expressed for a 1 interquartile range (i.e., 75$^{th}$ %tile - 25$^{th}$ %tile) change in ambient concentration for each pollutant (when air pollutants were estimated using a spatial statistical model: 13.1 μg/m$^3$ for $PM_1$, 10.0 μg/m$^3$ for $PM_{2.5}$, 13.8 μg/m$^3$ for $PM_{10}$, and 7.3 μg/m$^3$ for $NO_2$; when air pollutants were measured by local air monitoring stations: 30.6 μg/m$^3$ for $PM_{10}$, 23.4 μg/m$^3$ for $SO_2$, 13.0 μg/m$^3$ for $NO_2$, and 46.3 μg/m$^3$ for $O_3$).

© 2019 Zhang C et al. *JAMA Network Open*.

**eTable 5.** Crude Estimated Absolute Change in Lung Function Test Measurements Associated With 4-Year Mean Ambient Air Pollutant Concentrations Stratified by Breastfed Status

| Pollutant | Non-breastfed | Breastfed | Interaction |
|---|---|---|---|
| | β (95%CI)[a] | β (95%CI)[a] | *P* Value |
| FVC (mL) | | | |
| Air Pollutants estimated using a spatial statistical model | | | |
| $PM_1$, μg/$m^3$ | -198.47 (-261.51, -135.43) | -30.27 (-70.43, 9.90) | <0.001 |
| $PM_{2.5}$, μg/$m^3$ | -167.70 (-222.36, -113.04) | -35.03 (-69.87, -0.18) | 0.003 |
| $PM_{10}$, μg/$m^3$ | -141.14 (-190.55, -91.72) | -57.68 (-90.31, -25.05) | 0.156 |
| $NO_2$, μg/$m^3$ | -140.43 (-194.85, -86.01) | -101.01 (-137.87, -64.16) | 0.716 |
| Air Pollutants measured by local air monitoring station | | | |
| $PM_{10}$, μg/$m^3$ | -106.35 (-213.54, 0.84) | -44.29 (-123.37, 34.79) | <0.001 |
| $SO_2$, μg/$m^3$ | -42.97 (-159.78, 73.85) | -5.74 (-87.67, 76.18) | 0.070 |
| $NO_2$, μg/$m^3$ | -38.58 (-131.55, 54.40) | -2.19 (-68.20, 63.82) | 0.033 |
| $O_3$, μg/$m^3$ | -23.09 (-42.88, -3.30) | -8.00 (-23.24, 7.23) | 0.001 |
| $FEV_1$ (mL) | | | |
| Air Pollutants estimated using a spatial statistical model | | | |
| $PM_1$, μg/$m^3$ | -160.49 (-216.50, -104.48) | -20.91 (-56.29, 14.47) | <0.001 |
| $PM_{2.5}$, μg/$m^3$ | -136.05 (-184.54, -87.57) | -26.39 (-57.08, 4.31) | 0.002 |
| $PM_{10}$, μg/$m^3$ | -114.16 (-157.93, -70.39) | -44.96 (-73.72, -16.19) | 0.066 |
| $NO_2$, μg/$m^3$ | -113.96 (-162.19, -65.72) | -82.77 (-115.26, -50.29) | 0.945 |
| Air Pollutants measured by local air monitoring station | | | |
| $PM_{10}$, μg/$m^3$ | -97.78 (-186.61, -8.95) | -73.69 (-142.12, -5.27) | 0.003 |
| $SO_2$, μg/$m^3$ | -55.68 (-127.60, 16.23) | -33.52 (-132.00, 64.97) | 0.904 |
| $NO_2$, μg/$m^3$ | -25.56 (-104.16, 53.04) | -25.87 (-85.66, 33.93) | 0.257 |
| $O_3$, μg/$m^3$ | -17.92 (-34.84, -0.99) | -8.60 (-22.48, 5.29) | 0.002 |
| PEF (mL/s) | | | |
| Air Pollutants estimated using a spatial statistical model | | | |
| $PM_1$, μg/$m^3$ | -260.88 (-384.86, -136.91) | -45.56 (-123.47, 32.36) | 0.008 |
| $PM_{2.5}$, μg/$m^3$ | -190.75 (-297.89, -83.62) | -54.97 (-122.49, 12.55) | 0.036 |
| $PM_{10}$, μg/$m^3$ | -139.64 (-235.89, -43.39) | -76.63 (-139.71, -13.55) | 0.169 |
| $NO_2$, μg/$m^3$ | -132.98 (-238.79, -27.17) | -104.26 (-175.38, -33.14) | 0.460 |
| Air Pollutants measured by local air monitoring station | | | |
| $PM_{10}$, μg/$m^3$ | -234.11 (-410.48, -57.73) | -228.41 (-342.41, -114.41) | 0.202 |
| $SO_2$, μg/$m^3$ | -142.49 (-339.85, 54.87) | -115.75 (-254.71, 23.21) | 0.351 |
| $NO_2$, μg/$m^3$ | -90.63 (-249.92, 68.65) | -60.83 (-176.16, 54.49) | 0.253 |
| $O_3$, μg/$m^3$ | -32.07 (-67.69, 3.55) | -25.23 (-50.90, 0.43) | 0.283 |
| MMEF (mL/s) | | | |
| Air Pollutants estimated using a spatial statistical model | | | |
| $PM_1$, μg/$m^3$ | -73.29 (-161.66, 15.08) | -4.94 (-61.39, 51.50) | 0.196 |
| $PM_{2.5}$, μg/$m^3$ | -45.42 (-121.61, 30.76) | -17.05 (-66.01, 31.92) | 0.327 |
| $PM_{10}$, μg/$m^3$ | -27.22 (-95.50, 41.05) | -32.81 (-78.64, 13.03) | 0.561 |
| $NO_2$, μg/$m^3$ | -15.37 (-90.19, 59.45) | -51.84 (-103.51, -0.17) | 0.947 |
| Air Pollutants measured by local air monitoring station | | | |
| $PM_{10}$, μg/$m^3$ | -223.40 (-324.95, -121.86) | -200.07 (-280.93, -119.22) | 0.303 |
| $SO_2$, μg/$m^3$ | -177.86 (-269.14, -86.57) | -164.52 (-289.00, -40.03) | 0.914 |
| $NO_2$, μg/$m^3$ | -101.62 (-206.32, 3.08) | -72.70 (-162.83, 17.43) | 0.192 |

© 2019 Zhang C et al. *JAMA Network Open*.

| | | | |
|---|---|---|---|
| $O_3$, μg/$m^3$ | -28.45 (-51.53, -5.37) | -24.19 (-44.16, -4.23) | 0.426 |

Abbreviations: CI, confidence interval; $FEV_1$, forced expiratory volume in 1 second; FVC, forced vital capacity; MMEF, maximum mid expiratory flow rate; $O_3$, ozone; $NO_2$, nitrogen dioxide; PEF, peak expiratory flow rate; $PM_1$, airborne particulates with aerodynamic diameter ≤1 μm; $PM_{2.5}$, ≤2.5 μm, $PM_{10}$, ≤10 μm; $SO_2$, sulfur dioxide.

[a]Effect expressed for a 1 interquartile range (i.e., $75^{th}$ %tile - $25^{th}$ %tile) change in ambient concentration for each pollutant (when air pollutants were estimated using a spatial statistical model: 13.1 μg/$m^3$ for $PM_1$, 10.0 μg/$m^3$ for $PM_{2.5}$, 13.8 μg/$m^3$ for $PM_{10}$, and 7.3 μg/$m^3$ for $NO_2$; when air pollutants were measured by local air monitoring stations: 30.6 μg/$m^3$ for $PM_{10}$, 23.4 μg/$m^3$ for $SO_2$, 13.0 μg/$m^3$ for $NO_2$, and 46.3 μg/$m^3$ for $O_3$).

© 2019 Zhang C et al. *JAMA Network Open*.

**eTable 6.** Adjusted Odds Ratios (AORs) for Impaired Lung Function and 4-Year Mean Ambient Air Pollutant Concentrations Measured by Local Air Monitoring Stations Stratified by Breastfed Status

| Pollutant | Non-breastfed | Breastfed | Interaction |
|---|---|---|---|
| | AOR (95%CI) [ab] | AOR (95%CI) [ab] | *P*-Value |
| FVC < 85% of predicted value | | | |
| $PM_{10}$, μg/m$^3$ | 2.17 (1.69-2.79) | 1.32 (1.09-1.60) | <0.001 |
| $SO_2$, μg/m$^3$ | 1.59 (1.17-2.17) | 1.24 (0.97-1.60) | 0.096 |
| $NO_2$, μg/m$^3$ | 1.33 (1.04-1.71) | 1.07 (0.87-1.31) | 0.050 |
| $O_3$, μg/m$^3$ | 1.09 (1.03-1.14) | 1.03 (0.98-1.07) | 0.007 |
| FEV1 < 85% of predicted value | | | |
| $PM_{10}$, μg/m$^3$ | 2.25 (1.65-3.07) | 1.59 (1.23-2.05) | 0.022 |
| $SO_2$, μg/m$^3$ | 1.56 (1.14-2.15) | 1.34 (0.92-1.95) | 0.355 |
| $NO_2$, μg/m$^3$ | 1.32 (0.97-1.80) | 1.19 (0.91-1.55) | 0.385 |
| $O_3$, μg/m$^3$ | 1.08 (1.01-1.15) | 1.01 (0.95-1.08) | 0.013 |
| PEF < 75% of predicted value | | | |
| $PM_{10}$, μg/m$^3$ | 1.80 (1.30-2.47) | 1.69 (1.30-2.20) | 0.705 |
| $SO_2$, μg/m$^3$ | 1.51 (1.08-2.13) | 1.25 (0.84-1.87) | 0.314 |
| $NO_2$, μg/m$^3$ | 1.63 (1.19-2.23) | 1.26 (0.97-1.64) | 0.079 |
| $O_3$, μg/m$^3$ | 1.07 (1.00-1.14) | 1.04 (0.98-1.11) | 0.421 |
| MMEF < 75% of predicted value | | | |
| $PM_{10}$, μg/m$^3$ | 1.74 (1.29-2.35) | 1.68 (1.32-2.16) | 0.824 |
| $SO_2$, μg/m$^3$ | 1.73 (1.28-2.33) | 1.17 (0.82-1.67) | 0.021 |
| $NO_2$, μg/m$^3$ | 1.44 (1.09-1.92) | 1.30 (1.02-1.65) | 0.406 |
| $O_3$, μg/m$^3$ | 1.05 (0.98-1.12) | 1.04 (0.98-1.10) | 0.642 |

Abbreviations: CI, confidence interval; $FEV_1$, forced expiratory volume in 1 second; FVC, forced vital capacity; MMEF, maximum mid expiratory flow rate; $O_3$, ozone; $NO_2$, nitrogen dioxide; PEF, peak expiratory flow rate; $PM_{10}$, airborne particulates with aerodynamic diameter <10 μm; $SO_2$, sulfur dioxide.

[a]Adjusted for age, sex, height, birth weight, preterm birth, parental education, family income per year, exercise per week, passive smoke exposure, home coal use, house pet, home renovation in the past 2 years, area of residence per person, doctor-diagnosed asthma, family history of atopy, and short-term air pollution concentrations.

[b]Effect expressed for a 1 interquartile range (i.e., 75$^{th}$ %tile - 25$^{th}$ %tile) change in ambient concentration for each pollutant (30.6 μg/m$^3$ for $PM_{10}$, 23.4 μg/m$^3$ for $SO_2$, 13.0 μg/m$^3$ for $NO_2$, and 46.3 μg/m$^3$ for $O_3$).

© 2019 Zhang C et al. *JAMA Network Open*.

**eTable 7.** Estimated Absolute Change in Lung Function Test Measurements Associated With 4-Year Mean Ambient Air Pollutant Concentrations Measured by Local Air Monitoring Station Stratified by Breastfed Status

| Pollutant | Non-breastfed | Breastfed | Interaction |
|---|---|---|---|
| | β (95%CI) [ab] | β (95%CI) [ab] | *P*-Value |
| FVC (mL) | | | |
| $PM_{10}$, μg/$m^3$ | -90.53 (-141.17, -39.88) | -19.15 (-57.02, 18.73) | <0.001 |
| $SO_2$, μg/$m^3$ | -41.87 (-104.28, 20.54) | -4.18 (-43.24, 34.89) | 0.064 |
| $NO_2$, μg/$m^3$ | -25.51 (-75.17, 24.16) | 4.21 (-26.76, 35.18) | 0.072 |
| $O_3$, μg/$m^3$ | -14.44 (-24.23, -4.64) | -0.51 (-7.74, 6.73) | <0.001 |
| $FEV_1$ (mL) | | | |
| $PM_{10}$, μg/$m^3$ | -77.50 (-116.16, -38.84) | -48.20 (-96.13, -0.27) | 0.001 |
| $SO_2$, μg/$m^3$ | -48.18 (-96.46, 0.11) | -31.04 (-80.61, 18.53) | 0.854 |
| $NO_2$, μg/$m^3$ | -10.86 (-50.57, 28.85) | -18.03 (-59.38, 23.32) | 0.658 |
| $O_3$, μg/$m^3$ | -9.59 (-17.73, -1.44) | -0.01 (-9.91, 9.88) | <0.001 |
| PEF (mL/s) | | | |
| $PM_{10}$, μg/$m^3$ | -203.83 (-310.55, -97.12) | -195.24 (-269.21, -121.27) | 0.307 |
| $SO_2$, μg/$m^3$ | -138.63 (-269.56, -7.71) | -120.31 (-220.16, -20.45) | 0.480 |
| $NO_2$, μg/$m^3$ | -72.39 (-179.16, 34.38) | -51.17 (-137.97, 35.63) | 0.552 |
| $O_3$, μg/$m^3$ | -18.53 (-42.77, 5.72) | -13.40 (-33.66, 6.86) | 0.216 |
| MMEF (mL/s) | | | |
| $PM_{10}$, μg/$m^3$ | -179.59 (-274.03, -85.14) | -167.59 (-253.60, -81.59) | 0.370 |
| $SO_2$, μg/$m^3$ | -159.29 (-249.46, -69.13) | -142.09 (-251.48, -32.71) | 0.803 |
| $NO_2$, μg/$m^3$ | -82.92 (-174.32, 8.48) | -64.66 (-150.60, 21.27) | 0.411 |
| $O_3$, μg/$m^3$ | -18.39 (-39.72, 2.93) | -15.50 (-35.78, 4.79) | 0.422 |

Abbreviations: CI, confidence interval; $FEV_1$, forced expiratory volume in 1 second; FVC, forced vital capacity; MMEF, maximum mid expiratory flow rate; $O_3$, ozone; $NO_2$, nitrogen dioxide; PEF, peak expiratory flow rate; $PM_{10}$, airborne particulates with aerodynamic diameter ≤10 μm; $SO_2$, sulfur dioxide.

[a]Adjusted for age, sex, height, birth weight, preterm birth, parental education, family income per year, exercise per week, passive smoke exposure, home coal use, house pet, home renovation in the past 2 years, area of residence per person, doctor-diagnosed asthma, family history of atopy, and short-term air pollution concentrations.

[b]Effect expressed for a 1 interquartile range (i.e., $75^{th}$ %tile - $25^{th}$ %tile) change in ambient concentration for each pollutant (30.6 μg/$m^3$ for $PM_{10}$, 23.4 μg/$m^3$ for $SO_2$, 13.0 μg/$m^3$ for $NO_2$, and 46.3 μg/$m^3$ for $O_3$).

© 2019 Zhang C et al. *JAMA Network Open*.

**eTable 8.** Adjusted Odds Ratios (AORs) for Decreased Lung Function and 4-Year Mean Ambient Air Pollutant Concentrations Stratified by Breastfed Status and Excluding Children With Mixed Feeding

| Pollutant | Non-breastfed | Breastfed | Interaction |
|---|---|---|---|
| | AOR (95%CI) [ab] | AOR (95%CI) [ab] | *P*-Value |
| FVC < 85% of predicted value | | | |
| Air Pollutants estimated using a spatial statistical model | | | |
| $PM_1$, μg/$m^3$ | 2.76 (2.06-3.70) | 1.20 (0.97-1.50) | <0.001 |
| $PM_{2.5}$, μg/$m^3$ | 2.32 (1.82-2.94) | 1.26 (1.04-1.53) | <0.001 |
| $PM_{10}$, μg/$m^3$ | 1.97 (1.61-2.42) | 1.47 (1.23-1.75) | 0.018 |
| $NO_2$, μg/$m^3$ | 1.91 (1.51-2.40) | 1.69 (1.38-2.06) | 0.380 |
| Air Pollutants measured by local air monitoring station | | | |
| $PM_{10}$, μg/$m^3$ | 2.16 (1.69-2.76) | 1.30 (1.07-1.57) | <0.001 |
| $SO_2$, μg/$m^3$ | 1.59 (1.18-2.16) | 1.22 (0.96-1.57) | 0.080 |
| $NO_2$, μg/$m^3$ | 1.33 (1.04-1.69) | 1.06 (0.87-1.30) | 0.049 |
| $O_3$, μg/$m^3$ | 1.08 (1.03-1.14) | 1.02 (0.98-1.07) | 0.006 |
| FEV1 < 85% of predicted value | | | |
| Air Pollutants estimated using a spatial statistical model | | | |
| $PM_1$, μg/$m^3$ | 2.85 (2.05-3.97) | 1.49 (1.15-1.93) | 0.001 |
| $PM_{2.5}$, μg/$m^3$ | 2.71 (2.06-3.57) | 1.58 (1.27-1.98) | 0.001 |
| $PM_{10}$, μg/$m^3$ | 2.49 (1.97-3.15) | 1.85 (1.51-2.26) | 0.033 |
| $NO_2$, μg/$m^3$ | 2.66 (2.04-3.46) | 2.24 (1.78-2.82) | 0.285 |
| Air Pollutants measured by local air monitoring station | | | |
| $PM_{10}$, μg/$m^3$ | 2.22 (1.63-3.01) | 1.56 (1.21-2.02) | 0.022 |
| $SO_2$, μg/$m^3$ | 1.54 (1.12-2.12) | 1.32 (0.91-1.91) | 0.340 |
| $NO_2$, μg/$m^3$ | 1.31 (0.97-1.77) | 1.17 (0.89-1.52) | 0.362 |
| $O_3$, μg/$m^3$ | 1.08 (1.01-1.15) | 1.01 (0.95-1.08) | 0.012 |
| PEF < 75% of predicted value | | | |
| Air Pollutants estimated using a spatial statistical model | | | |
| $PM_1$, μg/$m^3$ | 1.76 (1.25-2.47) | 1.30 (0.99-1.72) | 0.156 |
| $PM_{2.5}$, μg/$m^3$ | 1.57 (1.18-2.09) | 1.23 (0.97-1.57) | 0.172 |
| $PM_{10}$, μg/$m^3$ | 1.43 (1.12-1.84) | 1.24 (0.99-1.54) | 0.330 |
| $NO_2$, μg/$m^3$ | 1.48 (1.12-1.95) | 1.35 (1.06-1.72) | 0.593 |
| Air Pollutants measured by local air monitoring station | | | |
| $PM_{10}$, μg/$m^3$ | 1.78 (1.29-2.46) | 1.70 (1.29-2.23) | 0.761 |
| $SO_2$, μg/$m^3$ | 1.49 (1.05-2.11) | 1.24 (0.82-1.86) | 0.360 |
| $NO_2$, μg/$m^3$ | 1.62 (1.18-2.23) | 1.23 (0.93-1.61) | 0.056 |
| $O_3$, μg/$m^3$ | 1.06 (0.99-1.14) | 1.04 (0.98-1.11) | 0.403 |
| MMEF < 75% of predicted value | | | |
| Air Pollutants estimated using a spatial statistical model | | | |
| $PM_1$, μg/$m^3$ | 1.43 (1.05-1.93) | 1.22 (0.97-1.55) | 0.412 |
| $PM_{2.5}$, μg/$m^3$ | 1.36 (1.06-1.75) | 1.22 (1.00-1.49) | 0.482 |
| $PM_{10}$, μg/$m^3$ | 1.33 (1.07-1.66) | 1.27 (1.06-1.53) | 0.732 |
| $NO_2$, μg/$m^3$ | 1.35 (1.05-1.73) | 1.37 (1.11-1.70) | 0.895 |
| Air Pollutants measured by local air monitoring station | | | |
| $PM_{10}$, μg/$m^3$ | 1.70 (1.27-2.28) | 1.69 (1.33-2.15) | 0.969 |
| $SO_2$, μg/$m^3$ | 1.71 (1.27-2.30) | 1.16 (0.81-1.65) | 0.023 |
| $NO_2$, μg/$m^3$ | 1.42 (1.07-1.88) | 1.29 (1.01-1.63) | 0.430 |

© 2019 Zhang C et al. *JAMA Network Open*.

| | | | |
|---|---|---|---|
| $O_3$, μg/$m^3$ | 1.05 (0.98-1.11) | 1.03 (0.97-1.09) | 0.566 |

Abbreviations: CI, confidence interval; $FEV_1$, forced expiratory volume in 1 second; FVC, forced vital capacity; MMEF, maximum mid expiratory flow rate; $O_3$, ozone; $NO_2$, nitrogen dioxides; PEF, peak expiratory flow rate; $PM_1$, airborne particulates with aerodynamic diameter <1 µm; $PM_{2.5}$, ≤2.5 µm, $PM_{10}$, ≤10 µm; $SO_2$, sulfur dioxide.

[a]Adjusted for age, sex, height, birth weight, preterm birth, parental education, family income per year, exercise per week, passive smoke exposure, home coal use, house pet, home renovation in the past 2 years, area of residence per person, doctor-diagnosed asthma, family history of atopy, and short-term air pollution concentrations.

[b]Effect expressed for a 1 interquartile range (i.e., $75^{th}$ %tile - $25^{th}$ %tile) change in ambient concentration for each pollutant (air pollutants were estimated using a spatial statistical model: 13.1 μg/$m^3$ for $PM_1$, 10.0 μg/$m^3$ for $PM_{2.5}$, 13.8 μg/$m^3$ for $PM_{10}$, 7.3 μg/$m^3$ for $NO_2$; air pollutants were measured by local air monitoring stations: 30.6 μg/$m^3$ for $PM_{10}$, 23.4 μg/$m^3$ for $SO_2$, 13.0 μg/$m^3$ for $NO_2$, and 46.3 μg/$m^3$ for $O_3$).

© 2019 Zhang C et al. *JAMA Network Open*.

**eTable 9.** Estimated Absolute Change in Lung Function Test Measurements Associated With 4-Year Mean Ambient Air Pollutant Concentrations Stratified by Breastfed Status and Excluding Children With Mixed Feeding

| Pollutant | Non-breastfed | Breastfed | Interaction |
|---|---|---|---|
| | β (95%CI) [ab] | β (95%CI) [ab] | *P*-Value |
| FVC (mL) | | | |
| Air Pollutants estimated using a spatial statistical model | | | |
| $PM_1$, μg/m$^3$ | -240.46 (-288.71, -192.21) | -39.68 (-71.74, -7.61) | <0.001 |
| $PM_{2.5}$, μg/m$^3$ | -217.97 (-259.95, -175.99) | -44.04 (-71.76, -16.32) | <0.001 |
| $PM_{10}$, μg/m$^3$ | -185.99 (-224.08, -147.89) | -58.21 (-83.98, -32.44) | 0.004 |
| $NO_2$, μg/m$^3$ | -179.26 (-221.32, -137.20) | -90.89 (-119.97, -61.82) | 0.439 |
| Air Pollutants measured by local air monitoring station | | | |
| $PM_{10}$, μg/m$^3$ | -90.53 (-141.17, -39.88) | -13.24 (-51.04, 24.57) | <0.001 |
| $SO_2$, μg/m$^3$ | -41.87 (-104.28, 20.54) | -3.27 (-42.15, 35.61) | 0.059 |
| $NO_2$, μg/m$^3$ | -25.51 (-75.17, 24.16) | 4.54 (-26.19, 35.28) | 0.086 |
| $O_3$, μg/m$^3$ | -14.44 (-24.23, -4.64) | -0.19 (-7.35, 6.97) | <0.001 |
| $FEV_1$ (mL) | | | |
| Air Pollutants estimated using a spatial statistical model | | | |
| $PM_1$, μg/m$^3$ | -201.37 (-242.08, -160.65) | -31.63 (-59.86, -3.40) | <0.001 |
| $PM_{2.5}$, μg/m$^3$ | -181.68 (-217.06, -146.30) | -35.15 (-59.70, -10.61) | <0.001 |
| $PM_{10}$, μg/m$^3$ | -154.14 (-186.37, -121.90) | -49.14 (-72.09, -26.18) | <0.001 |
| $NO_2$, μg/m$^3$ | -150.26 (-186.14, -114.39) | -80.50 (-106.25, -54.75) | 0.176 |
| Air Pollutants measured by local air monitoring station | | | |
| $PM_{10}$, μg/m$^3$ | -77.50 (-116.16, -38.84) | -42.69 (-89.69, 4.31) | 0.001 |
| $SO_2$, μg/m$^3$ | -48.18 (-96.46, 0.11) | -27.96 (-77.35, 15.41) | 0.751 |
| $NO_2$, μg/m$^3$ | -10.86 (-50.57, 28.85) | -17.31 (-57.25, 22.63) | 0.713 |
| $O_3$, μg/m$^3$ | -9.59 (-17.73, -1.44) | 0.43 (-9.09, 9.95) | <0.001 |
| PEF (mL/s) | | | |
| Air Pollutants estimated using a spatial statistical model | | | |
| $PM_1$, μg/m$^3$ | -333.29 (-438.21, -228.37) | -52.83 (-122.16, 16.49) | <0.001 |
| $PM_{2.5}$, μg/m$^3$ | -273.82 (-363.94, -183.69) | -59.41 (-119.58, 0.76) | <0.001 |
| $PM_{10}$, μg/m$^3$ | -214.86 (-295.23, -134.48) | -70.50 (-126.52, -14.49) | 0.006 |
| $NO_2$, μg/m$^3$ | -206.06 (-294.94, -117.18) | -85.77 (-148.60, -22.95) | 0.044 |
| Air Pollutants measured by local air monitoring station | | | |
| $PM_{10}$, μg/m$^3$ | -203.83 (-310.55, -97.12) | -190.55 (-264.10, -117.01) | 0.280 |
| $SO_2$, μg/m$^3$ | -138.63 (-269.56, -7.71) | -107.49 (-207.32, -7.67) | 0.337 |
| $NO_2$, μg/m$^3$ | -72.39 (-179.16, 34.38) | -47.93 (-133.21, 37.34) | 0.546 |
| $O_3$, μg/m$^3$ | -18.53 (-42.77, 5.72) | -12.29 (-32.14, 7.56) | 0.207 |
| MMEF (mL/s) | | | |
| Air Pollutants estimated using a spatial statistical model | | | |
| $PM_1$, μg/m$^3$ | -127.42 (-208.01, -46.84) | -5.89 (-59.51, 47.72) | 0.022 |
| $PM_{2.5}$, μg/m$^3$ | -101.86 (-171.22, -32.49) | -17.26 (-63.94, 29.43) | 0.045 |
| $PM_{10}$, μg/m$^3$ | -77.17 (-139.14, -15.19) | -30.91 (-74.66, 12.83) | 0.161 |
| $NO_2$, μg/m$^3$ | -66.16 (-134.19, 1.87) | -45.35 (-94.42, 3.71) | 0.542 |
| Air Pollutants measured by local air monitoring station | | | |
| $PM_{10}$, μg/m$^3$ | -179.59 (-274.03, -85.14) | -165.04 (-246.81, -83.27) | 0.383 |
| $SO_2$, μg/m$^3$ | -159.29 (-249.46, -69.13) | -138.92 (-235.83, -41.60) | 0.702 |
| $NO_2$, μg/m$^3$ | -82.92 (-174.32, 8.48) | -60.90 (-144.00, 22.20) | 0.397 |
| $O_3$, μg/m$^3$ | -18.39 (-39.72, 2.93) | -14.00 (-33.64, 5.64) | 0.371 |

© 2019 Zhang C et al. *JAMA Network Open*.

Abbreviations: CI, confidence interval; $FEV_1$, forced expiratory volume in 1 second; FVC, forced vital capacity; MMEF, maximum mid expiratory flow rate; $O_3$, ozone; $NO_2$, nitrogen dioxides; PEF, peak expiratory flow rate; $PM_1$, airborne particulates with aerodynamic diameter ≤1 µm; $PM_{2.5}$, ≤2.5 µm, $PM_{10}$, ≤10 µm; $SO_2$, sulfur dioxide.
[a]Adjusted for age, sex, height, birth weight, preterm birth, parental education, family income per year, exercise per week, passive smoke exposure, home coal use, house pet, home renovation in the past 2 years, area of residence per person, doctor-diagnosed asthma, family history of atopy, and short-term air pollution concentrations.
[b]Effect expressed for a 1 interquartile range (i.e., 75$^{th}$ %tile - 25$^{th}$ %tile) change in ambient concentration for each pollutant (air pollutants were estimated using a spatial statistical model: 13.1 µg/m$^3$ for $PM_1$, 10.0 µg/m$^3$ for $PM_{2.5}$, 13.8 µg/m$^3$ for $PM_{10}$, 7.3 µg/m$^3$ for $NO_2$; air pollutants were measured by local air monitoring stations: 30.6 µg/m$^3$ for $PM_{10}$, 23.4 µg/m$^3$ for $SO_2$, 13.0 µg/m$^3$ for $NO_2$, and 46.3 µg/m$^3$ for $O_3$).

© 2019 Zhang C et al. *JAMA Network Open*.

**eTable 10.** Adjusted Odds Ratios (AORs) for Impaired Lung Function and 4-Year Mean Ambient Air Pollutant Concentrations Stratified by Breastfed Status and Excluding Children With Low Birth Weight or Preterm Birth

| Pollutant | Non-breastfed | Breastfed | Interaction |
|---|---|---|---|
| | OR (95%CI) [ab] | OR (95%CI) [ab] | *P*-Value |
| FVC < 85% of predicted value | | | |
| Air Pollutants estimated using a spatial statistical model | | | |
| $PM_1$, µg/m$^3$ | 2.59 (1.90-3.53) | 1.19 (0.95-1.47) | <0.001 |
| $PM_{2.5}$, µg/m$^3$ | 2.22 (1.73-2.86) | 1.26 (1.04-1.52) | <0.001 |
| $PM_{10}$, µg/m$^3$ | 1.94 (1.57-2.40) | 1.47 (1.24-1.76) | 0.032 |
| $NO_2$, µg/m$^3$ | 1.91 (1.50-2.44) | 1.70 (1.39-2.07) | 0.406 |
| Air Pollutants measured by local air monitoring station | | | |
| $PM_{10}$, µg/m$^3$ | 2.15 (1.65-2.80) | 1.34 (1.10-1.63) | 0.001 |
| $SO_2$, µg/m$^3$ | 1.56 (1.12-2.16) | 1.21 (0.93-1.57) | 0.109 |
| $NO_2$, µg/m$^3$ | 1.46 (1.13-1.89) | 1.10 (0.90-1.35) | 0.020 |
| $O_3$, µg/m$^3$ | 1.09 (1.04-1.15) | 1.03 (0.98-1.08) | 0.004 |
| FEV1 < 85% of predicted value | | | |
| Air Pollutants estimated using a spatial statistical model | | | |
| $PM_1$, µg/m$^3$ | 2.74 (1.93-3.87) | 1.44 (1.11-1.85) | 0.002 |
| $PM_{2.5}$, µg/m$^3$ | 2.66 (2.00-3.55) | 1.55 (1.24-1.93) | 0.002 |
| $PM_{10}$, µg/m$^3$ | 2.48 (1.94-3.17) | 1.83 (1.49-2.23) | 0.034 |
| $NO_2$, µg/m$^3$ | 2.67 (2.02-3.52) | 2.19 (1.74-2.75) | 0.225 |
| Air Pollutants measured by local air monitoring station | | | |
| $PM_{10}$, µg/m$^3$ | 2.22 (1.61-3.06) | 1.62 (1.25-2.11) | 0.047 |
| $SO_2$, µg/m$^3$ | 1.56 (1.12-2.15) | 1.37 (0.93-2.01) | 0.464 |
| $NO_2$, µg/m$^3$ | 1.37 (1.00-1.88) | 1.21 (0.92-1.59) | 0.345 |
| $O_3$, µg/m$^3$ | 1.08 (1.01-1.16) | 1.01 (0.95-1.08) | 0.009 |
| PEF < 75% of predicted value | | | |
| Air Pollutants estimated using a spatial statistical model | | | |
| $PM_1$, µg/m$^3$ | 1.77 (1.23-2.55) | 1.24 (0.94-1.64) | 0.106 |
| $PM_{2.5}$, µg/m$^3$ | 1.55 (1.14-2.10) | 1.18 (0.93-1.50) | 0.138 |
| $PM_{10}$, µg/m$^3$ | 1.41 (1.09-1.83) | 1.18 (0.95-1.47) | 0.260 |
| $NO_2$, µg/m$^3$ | 1.46 (1.09-1.95) | 1.29 (1.02-1.64) | 0.492 |
| Air Pollutants measured by local air monitoring station | | | |
| $PM_{10}$, µg/m$^3$ | 1.65 (1.18-2.30) | 1.68 (1.29-2.21) | 0.894 |
| $SO_2$, µg/m$^3$ | 1.55 (1.10-2.17) | 1.21 (0.80-1.83) | 0.220 |
| $NO_2$, µg/m$^3$ | 1.60 (1.15-2.22) | 1.25 (0.96-1.64) | 0.119 |
| $O_3$, µg/m$^3$ | 1.06 (0.99-1.14) | 1.04 (0.98-1.11) | 0.446 |
| MMEF < 75% of predicted value | | | |
| Air Pollutants estimated using a spatial statistical model | | | |
| $PM_1$, µg/m$^3$ | 1.45 (1.05-2.01) | 1.23 (0.98-1.56) | 0.394 |
| $PM_{2.5}$, µg/m$^3$ | 1.41 (1.08-1.84) | 1.25 (1.02-1.52) | 0.453 |
| $PM_{10}$, µg/m$^3$ | 1.41 (1.11-1.78) | 1.31 (1.08-1.57) | 0.590 |
| $NO_2$, µg/m$^3$ | 1.48 (1.14-1.93) | 1.41 (1.14-1.75) | 0.758 |
| Air Pollutants measured by local air monitoring station | | | |
| $PM_{10}$, µg/m$^3$ | 1.61 (1.16-2.23) | 1.73 (1.33-2.26) | 0.636 |
| $SO_2$, µg/m$^3$ | 1.80 (1.32-2.46) | 1.09 (0.75-1.60) | 0.006 |

© 2019 Zhang C et al. *JAMA Network Open*.

| | | | |
|---|---|---|---|
| $NO_2$, µg/m$^3$ | 1.43 (1.05-1.94) | 1.30 (1.01-1.68) | 0.490 |
| $O_3$, µg/m$^3$ | 1.05 (0.98-1.12) | 1.04 (0.97-1.10) | 0.588 |

Abbreviations: CI, confidence interval; $FEV_1$, forced expiratory volume in 1 second; FVC, forced vital capacity; MMEF, maximum mid expiratory flow rate; $O_3$, ozone; $NO_2$, nitrogen dioxides; PEF, peak expiratory flow rate; $PM_1$, airborne particulates with aerodynamic diameter <1 µm; $PM_{2.5}$, ≤2.5 µm, $PM_{10}$, ≤10 µm; $SO_2$, sulfur dioxide.

[a]Adjusted for age, sex, height, birth weight, parental education, family income per year, exercise per week, passive smoke exposure, home coal use, house pet, home renovation in the past 2 years, area of residence per person, doctor-diagnosed asthma, family history of atopy, and short-term air pollution concentrations.

[b]Effect expressed for a 1 interquartile range (i.e., 75$^{th}$ %tile - 25$^{th}$ %tile) change in ambient concentration for each pollutant (air pollutants were estimated using a spatial statistical model: 13.1 µg/m$^3$ for $PM_1$, 10.0 µg/m$^3$ for $PM_{2.5}$, 13.8 µg/m$^3$ for $PM_{10}$, 7.3 µg/m$^3$ for $NO_2$; air pollutants were measured by local air monitoring stations: 30.6 µg/m$^3$ for $PM_{10}$, 23.4 µg/m$^3$ for $SO_2$, 13.0 µg/m$^3$ for $NO_2$, and 46.3 µg/m$^3$ for $O_3$).

© 2019 Zhang C et al. *JAMA Network Open*.

**eTable 11.** Estimated Absolute Change in Lung Function Test Measurements Associated With 4-Year Mean Ambient Air Pollutant Concentrations Stratified by Breastfed Status and Excluding Children With Low Birth Weight or Preterm Birth

| Pollutant | Non-breastfed | Breastfed | Interaction |
|---|---|---|---|
| | β (95%CI) [ab] | β (95%CI) [ab] | *P*-Value |
| FVC (mL) | | | |
| Air Pollutants estimated using a spatial statistical model | | | |
| $PM_1$, µg/m$^3$ | -237.14 (-288.06, -186.22) | -36.49 (-68.71, -4.27) | <0.001 |
| $PM_{2.5}$, µg/m$^3$ | -214.38 (-258.57, -170.20) | -40.11 (-67.91, -12.31) | <0.001 |
| $PM_{10}$, µg/m$^3$ | -182.61 (-222.53, -142.69) | -55.31 (-81.12, -29.50) | 0.005 |
| $NO_2$, µg/m$^3$ | -174.58 (-218.52, -130.64) | -87.84 (-117.01, -58.68) | 0.440 |
| Air Pollutants measured by local air monitoring station | | | |
| $PM_{10}$, µg/m$^3$ | -91.36 (-143.89, -38.82) | -22.05 (-62.16, 18.07) | <0.001 |
| $SO_2$, µg/m$^3$ | -38.63 (-103.13, 25.87) | -0.40 (-41.88, 41.09) | 0.069 |
| $NO_2$, µg/m$^3$ | -34.07 (-84.60, 16.46) | 1.74 (-31.22, 34.69) | 0.025 |
| $O_3$, µg/m$^3$ | -16.79 (-26.34, -7.24) | -0.97 (-8.64, 6.70) | <0.001 |
| $FEV_1$ (mL) | | | |
| Air Pollutants estimated using a spatial statistical model | | | |
| $PM_1$, µg/m$^3$ | -198.29 (-241.13, -155.44) | -26.22 (-54.51, 2.07) | <0.001 |
| $PM_{2.5}$, µg/m$^3$ | -179.05 (-216.16, -141.95) | -30.28 (-54.80, -5.76) | <0.001 |
| $PM_{10}$, µg/m$^3$ | -152.24 (-185.93, -118.55) | -45.87 (-68.78, -22.96) | <0.001 |
| $NO_2$, µg/m$^3$ | -148.74 (-186.14, -111.34) | -77.29 (-103.10, -51.48) | 0.154 |
| Air Pollutants measured by local air monitoring station | | | |
| $PM_{10}$, µg/m$^3$ | -78.63 (-119.07, -38.20) | -49.05 (-98.45, 0.35) | 0.001 |
| $SO_2$, µg/m$^3$ | -45.92 (-96.08, 4.24) | -30.74 (-82.20, 20.71) | 0.862 |
| $NO_2$, µg/m$^3$ | -19.13 (-59.95, 21.68) | -18.42 (-60.86, 24.02) | 0.305 |
| $O_3$, µg/m$^3$ | -12.31 (-20.16, -4.46) | 0.27 (-9.88, 10.41) | <0.001 |
| PEF (mL/s) | | | |
| Air Pollutants estimated using a spatial statistical model | | | |
| $PM_1$, µg/m$^3$ | -332.96 (-443.30, -222.61) | -55.23 (-124.61, 14.16) | <0.001 |
| $PM_{2.5}$, µg/m$^3$ | -270.95 (-365.38, -176.53) | -60.34 (-120.40, -0.27) | 0.001 |
| $PM_{10}$, µg/m$^3$ | -211.08 (-294.86, -127.29) | -72.84 (-128.78, -16.89) | 0.013 |
| $NO_2$, µg/m$^3$ | -206.51 (-298.96, -114.06) | -85.36 (-148.41, -22.32) | 0.050 |
| Air Pollutants measured by local air monitoring station | | | |
| $PM_{10}$, µg/m$^3$ | -184.87 (-298.68, -71.07) | -204.53 (-283.55, -125.52) | 0.618 |
| $SO_2$, µg/m$^3$ | -128.50 (-264.25, 7.24) | -127.25 (-232.71, -21.78) | 0.799 |
| $NO_2$, µg/m$^3$ | -92.43 (-199.30, 14.44) | -54.40 (-146.10, 37.30) | 0.321 |
| $O_3$, µg/m$^3$ | -22.57 (-46.55, 1.40) | -13.45 (-34.96, 8.07) | 0.073 |
| MMEF (mL/s) | | | |
| Air Pollutants estimated using a spatial statistical model | | | |
| $PM_1$, µg/m$^3$ | -129.49 (-214.66, -44.32) | -7.10 (-60.96, 46.76) | 0.037 |
| $PM_{2.5}$, µg/m$^3$ | -103.48 (-176.54, -30.42) | -17.74 (-64.48, 29.01) | 0.065 |
| $PM_{10}$, µg/m$^3$ | -80.24 (-145.23, -15.25) | -31.99 (-75.77, 11.78) | 0.168 |
| $NO_2$, µg/m$^3$ | -74.63 (-145.80, -3.45) | -47.22 (-96.58, 2.14) | 0.433 |
| Air Pollutants measured by local air monitoring station | | | |
| $PM_{10}$, µg/m$^3$ | -165.65 (-268.44, -62.87) | -172.24 (-259.86, -84.61) | 0.781 |
| $SO_2$, µg/m$^3$ | -161.53 (-253.89, -69.18) | -141.67 (-255.03, -28.31) | 0.580 |
| $NO_2$, µg/m$^3$ | -93.44 (-185.89, -0.99) | -64.29 (-152.23, 23.65) | 0.275 |

© 2019 Zhang C et al. *JAMA Network Open*.

| | | | |
|---|---|---|---|
| $O_3$, μg/$m^3$ | -20.33 (-41.82, 1.16) | -15.17 (-35.95, 5.61) | 0.246 |

Abbreviations: CI, confidence interval; $FEV_1$, forced expiratory volume in 1 second; FVC, forced vital capacity; MMEF, maximum mid expiratory flow rate; $O_3$, ozone; $NO_2$, nitrogen dioxides; PEF, peak expiratory flow rate; $PM_1$, airborne particulates with aerodynamic diameter ≤1 µm; $PM_{2.5}$, ≤2.5 µm, $PM_{10}$, ≤10 µm; $SO_2$, sulfur dioxide.

[a]Adjusted for age, sex, height, birth weight, parental education, family income per year, exercise per week, passive smoke exposure, home coal use, house pet, home renovation in the past 2 years, area of residence per person, doctor-diagnosed asthma, family history of atopy, and short-term air pollution concentrations.

[b]Effect expressed for a 1 interquartile range (i.e., $75^{th}$ %tile - $25^{th}$ %tile) change in ambient concentration for each pollutant (when air pollutants were estimated using a spatial statistical model: 13.1 μg/$m^3$ for $PM_1$, 10.0 μg/$m^3$ for $PM_{2.5}$, 13.8 μg/$m^3$ for $PM_{10}$, 7.3 μg/$m^3$ for $NO_2$; when air pollutants were measured by local air monitoring station: 30.6 μg/$m^3$ for $PM_{10}$, 23.4 μg/$m^3$ for $SO_2$, 13.0 μg/$m^3$ for $NO_2$, and 46.3 μg/$m^3$ for $O_3$).

© 2019 Zhang C et al. *JAMA Network Open*.

**eTable 12.** Adjusted Odds Ratios (AORs) for Impaired Lung Function and 4-Year Mean Ambient Air Pollutant Concentrations Stratified by Breastfed Status and Excluding Children With Asthma Diagnosed by a Physician

| Pollutant | Non-breastfed | Breastfed | Interaction |
|---|---|---|---|
| | OR (95%CI) [ab] | OR (95%CI) [ab] | *P*-Value |
| FVC < 85% of predicted value | | | |
| Air Pollutants estimated using a spatial statistical model | | | |
| $PM_1$, μg/m$^3$ | 2.50 (1.84-3.38) | 1.25 (1.00-1.56) | <0.001 |
| $PM_{2.5}$, μg/m$^3$ | 2.15 (1.68-2.76) | 1.30 (1.08-1.58) | 0.001 |
| $PM_{10}$, μg/m$^3$ | 1.89 (1.53-2.33) | 1.50 (1.26-1.79) | 0.073 |
| $NO_2$, μg/m$^3$ | 1.83 (1.44-2.32) | 1.73 (1.41-2.11) | 0.692 |
| Air Pollutants measured by local air monitoring station | | | |
| $PM_{10}$, μg/m$^3$ | 2.14 (1.64-2.79) | 1.31 (1.07-1.60) | 0.001 |
| $SO_2$, μg/m$^3$ | 1.64 (1.20-2.25) | 1.23 (0.96-1.59) | 0.067 |
| $NO_2$, μg/m$^3$ | 1.37 (1.06-1.77) | 1.06 (0.86-1.31) | 0.029 |
| $O_3$, μg/m$^3$ | 1.09 (1.03-1.14) | 1.03 (0.98-1.07) | 0.011 |
| FEV1 < 85% of predicted value | | | |
| Air Pollutants estimated using a spatial statistical model | | | |
| $PM_1$, μg/m$^3$ | 2.75 (1.95-3.88) | 1.49 (1.15-1.93) | 0.003 |
| $PM_{2.5}$, μg/m$^3$ | 2.59 (1.95-3.45) | 1.58 (1.27-1.98) | 0.004 |
| $PM_{10}$, μg/m$^3$ | 2.39 (1.88-3.05) | 1.84 (1.50-2.25) | 0.068 |
| $NO_2$, μg/m$^3$ | 2.53 (1.92-3.34) | 2.20 (1.74-2.78) | 0.394 |
| Air Pollutants measured by local air monitoring station | | | |
| $PM_{10}$, μg/m$^3$ | 2.14 (1.55-2.94) | 1.58 (1.22-2.05) | 0.057 |
| $SO_2$, μg/m$^3$ | 1.52 (1.11-2.09) | 1.37 (0.94-1.98) | 0.536 |
| $NO_2$, μg/m$^3$ | 1.33 (0.98-1.81) | 1.18 (0.91-1.54) | 0.375 |
| $O_3$, μg/m$^3$ | 1.07 (1.00-1.15) | 1.02 (0.96-1.08) | 0.047 |
| PEF < 75% of predicted value | | | |
| Air Pollutants estimated using a spatial statistical model | | | |
| $PM_1$, μg/m$^3$ | 1.82 (1.27-2.59) | 1.31 (0.99-1.73) | 0.128 |
| $PM_{2.5}$, μg/m$^3$ | 1.63 (1.21-2.19) | 1.24 (0.98-1.59) | 0.137 |
| $PM_{10}$, μg/m$^3$ | 1.51 (1.17-1.95) | 1.23 (0.99-1.54) | 0.193 |
| $NO_2$, μg/m$^3$ | 1.60 (1.20-2.13) | 1.34 (1.05-1.72) | 0.320 |
| Air Pollutants measured by local air monitoring station | | | |
| $PM_{10}$, μg/m$^3$ | 1.77 (1.28-2.45) | 1.66 (1.28-2.16) | 0.690 |
| $SO_2$, μg/m$^3$ | 1.48 (1.05-2.08) | 1.21 (0.81-1.81) | 0.299 |
| $NO_2$, μg/m$^3$ | 1.62 (1.18-2.22) | 1.24 (0.95-1.61) | 0.075 |
| $O_3$, μg/m$^3$ | 1.06 (1.00-1.14) | 1.04 (0.98-1.11) | 0.493 |
| MMEF < 75% of predicted value | | | |
| Air Pollutants estimated using a spatial statistical model | | | |
| $PM_1$, μg/m$^3$ | 1.41 (1.03-1.93) | 1.30 (1.03-1.65) | 0.672 |
| $PM_{2.5}$, μg/m$^3$ | 1.37 (1.06-1.79) | 1.31 (1.06-1.60) | 0.756 |
| $PM_{10}$, μg/m$^3$ | 1.36 (1.08-1.71) | 1.34 (1.11-1.62) | 0.923 |
| $NO_2$, μg/m$^3$ | 1.41 (1.09-1.83) | 1.44 (1.16-1.79) | 0.883 |
| Air Pollutants measured by local air monitoring station | | | |
| $PM_{10}$, μg/m$^3$ | 1.54 (1.13-2.11) | 1.63 (1.27-2.10) | 0.719 |
| $SO_2$, μg/m$^3$ | 1.65 (1.23-2.23) | 1.08 (0.76-1.56) | 0.017 |

© 2019 Zhang C et al. *JAMA Network Open*.

| | | | |
|---|---|---|---|
| $NO_2$, μg/$m^3$ | 1.40 (1.06-1.86) | 1.29 (1.02-1.62) | 0.512 |
| $O_3$, μg/$m^3$ | 1.05 (0.99-1.12) | 1.04 (0.98-1.10) | 0.712 |

Abbreviations: CI, confidence interval; $FEV_1$, forced expiratory volume in 1 second; FVC, forced vital capacity; MMEF, maximum mid expiratory flow rate; $O_3$, ozone; $NO_2$, nitrogen dioxides; PEF, peak expiratory flow rate; $PM_1$, airborne particulates with aerodynamic diameter <1 μm; $PM_{2.5}$, ≤2.5 μm, $PM_{10}$, ≤10 μm; $SO_2$, sulfur dioxide.

[a]Adjusted for age, sex, height, birth weight, preterm birth, parental education, family income per year, exercise per week, passive smoke exposure, home coal use, house pet, home renovation in the past 2 years, area of residence per person, family history of atopy, and short-term air pollution concentrations.

[b]Effect expressed for a 1 interquartile range (i.e., $75^{th}$ %tile - $25^{th}$ %tile) change in ambient concentration for each pollutant (when air pollutants were estimated using a spatial statistical model:13.1 μg/$m^3$ for $PM_1$, 10.0 μg/$m^3$ for $PM_{2.5}$, 13.8 μg/$m^3$ for $PM_{10}$, 7.3 μg/$m^3$ for $NO_2$; when air pollutants were measured by local air monitoring station: 30.6 μg/$m^3$ for $PM_{10}$, 23.4 μg/$m^3$ for $SO_2$, 13.0 μg/$m^3$ for $NO_2$, and 46.3 μg/$m^3$ for $O_3$).

© 2019 Zhang C et al. *JAMA Network Open*.

**eTable 13.** Estimated Absolute Change in Lung Function Test Measurements Associated With 4-Year Mean Ambient Air Pollutant Concentrations Stratified by Breastfed Status and Excluding Children With Asthma Diagnosed by a Physician

| Pollutant | Non-breastfed | Breastfed | Interaction |
|---|---|---|---|
| | β (95%CI) [ab] | β (95%CI) [ab] | *P*-Value |
| FVC (mL) | | | |
| Air Pollutants estimated using a spatial statistical model | | | |
| $PM_1$, μg/m$^3$ | -232.39 (-281.88, -182.91) | -48.66 (-81.04, -16.28) | <0.001 |
| $PM_{2.5}$, μg/m$^3$ | -207.15 (-250.22, -164.09) | -50.26 (-78.25, -22.27) | <0.001 |
| $PM_{10}$, μg/m$^3$ | -174.36 (-213.40, -135.31) | -62.50 (-88.54, -36.46) | 0.013 |
| $NO_2$, μg/m$^3$ | -165.10 (-208.11, -122.09) | -93.80 (-123.15, -64.45) | 0.590 |
| Air Pollutants measured by local air monitoring station | | | |
| $PM_{10}$, μg/m$^3$ | -87.20 (-139.81, -34.59) | -21.32 (-60.45, 17.80) | <0.001 |
| $SO_2$, μg/m$^3$ | -39.26 (-103.04, 24.52) | -7.14 (-47.43, 33.16) | 0.120 |
| $NO_2$, μg/m$^3$ | -29.84 (-80.02, 20.34) | 4.29 (-27.68, 36.25) | 0.045 |
| $O_3$, μg/m$^3$ | -14.82 (-24.76, -4.88) | -0.98 (-8.45, 6.49) | <0.001 |
| $FEV_1$ (mL) | | | |
| Air Pollutants estimated using a spatial statistical model | | | |
| $PM_1$, μg/m$^3$ | -193.84 (-235.44, -152.24) | -39.72 (-68.26, -11.18) | <0.001 |
| $PM_{2.5}$, μg/m$^3$ | -172.75 (-208.90, -136.60) | -41.35 (-66.14, -16.56) | <0.001 |
| $PM_{10}$, μg/m$^3$ | -144.39 (-177.33, -111.46) | -53.76 (-76.94, -30.58) | 0.002 |
| $NO_2$, μg/m$^3$ | -137.67 (-174.23, -101.12) | -83.69 (-109.71, -57.66) | 0.299 |
| Air Pollutants measured by local air monitoring station | | | |
| $PM_{10}$, μg/m$^3$ | -72.49 (-112.57, -32.41) | -47.70 (-96.51, 1.11) | 0.003 |
| $SO_2$, μg/m$^3$ | -48.76 (-97.71, 0.19) | -27.52 (-77.34, 22.30) | 0.744 |
| $NO_2$, μg/m$^3$ | -14.70 (-54.00, 24.60) | -16.27 (-58.25, 25.72) | 0.450 |
| $O_3$, μg/m$^3$ | -9.80 (-17.89, -1.71) | -0.39 (-10.40, 9.62) | <0.001 |
| PEF (mL/s) | | | |
| Air Pollutants estimated using a spatial statistical model | | | |
| $PM_1$, μg/m$^3$ | -334.21 (-442.95, -225.46) | -73.08 (-143.07, -3.09) | 0.001 |
| $PM_{2.5}$, μg/m$^3$ | -267.83 (-361.17, -174.49) | -78.53 (-139.22, -17.84) | 0.005 |
| $PM_{10}$, μg/m$^3$ | -203.92 (-287.01, -120.83) | -85.97 (-142.50, -29.43) | 0.039 |
| $NO_2$, μg/m$^3$ | -191.41 (-283.09, -99.73) | -96.51 (-160.02, -33.00) | 0.121 |
| Air Pollutants measured by local air monitoring station | | | |
| $PM_{10}$, μg/m$^3$ | -209.52 (-312.20, -106.84) | -195.79 (-273.32, -118.25) | 0.249 |
| $SO_2$, μg/m$^3$ | -140.96 (-268.71, -13.20) | -119.31 (-222.84, -15.78) | 0.387 |
| $NO_2$, μg/m$^3$ | -93.13 (-194.70, 8.45) | -47.70 (-137.63, 42.24) | 0.245 |
| $O_3$, μg/m$^3$ | -21.01 (-44.29, 2.27) | -14.55 (-35.28, 6.18) | 0.153 |
| MMEF (mL/s) | | | |
| Air Pollutants estimated using a spatial statistical model | | | |
| $PM_1$, μg/m$^3$ | -118.73 (-201.84, -35.62) | -28.12 (-82.18, 25.93) | 0.112 |
| $PM_{2.5}$, μg/m$^3$ | -93.54 (-164.97, -22.11) | -36.19 (-83.22, 10.83) | 0.199 |
| $PM_{10}$, μg/m$^3$ | -68.09 (-131.76, -4.42) | -45.69 (-89.73, -1.65) | 0.442 |
| $NO_2$, μg/m$^3$ | -54.75 (-124.57, 15.08) | -57.88 (-107.35, -8.41) | 0.878 |
| Air Pollutants measured by local air monitoring station | | | |
| $PM_{10}$, μg/m$^3$ | -164.00 (-257.22, -70.78) | -163.92 (-252.60, -75.25) | 0.527 |
| $SO_2$, μg/m$^3$ | -153.34 (-247.04, -59.65) | -128.61 (-234.59, -22.62) | 0.760 |
| $NO_2$, μg/m$^3$ | -85.94 (-171.44, -0.44) | -58.97 (-146.65, 28.70) | 0.287 |

© 2019 Zhang C et al. *JAMA Network Open*.

| | | | |
|---|---|---|---|
| $O_3$, μg/$m^3$ | -18.74 (-38.70, 1.23) | -16.01 (-36.48, 4.46) | 0.433 |

Abbreviations: CI, confidence interval; $FEV_1$, forced expiratory volume in 1 second; FVC, forced vital capacity; MMEF, maximum mid expiratory flow rate; $O_3$, ozone; $NO_2$, nitrogen dioxides; PEF, peak expiratory flow rate; $PM_1$, airborne particulates with aerodynamic diameter ≤1 µm; $PM_{2.5}$, ≤2.5 µm, $PM_{10}$, ≤10 µm; $SO_2$, sulfur dioxide.

[a]Adjusted for age, sex, height, birth weight, preterm birth, parental education, family income per year, exercise per week, passive smoke exposure, home coal use, house pet, home renovation in the past 2 years, area of residence per person, family history of atopy, and short-term air pollution concentrations.

[b]Effect expressed for a 1 interquartile range (i.e., $75^{th}$ %tile - $25^{th}$ %tile) change in ambient concentration for each pollutant (when air pollutants were estimated using a spatial statistical model: 13.1 μg/$m^3$ for $PM_1$, 10.0 μg/$m^3$ for $PM_{2.5}$, 13.8 μg/$m^3$ for $PM_{10}$, 7.3 μg/$m^3$ for $NO_2$; when air pollutants were measured by local air monitoring stations: 30.6 μg/$m^3$ for $PM_{10}$, 23.4 μg/$m^3$ for $SO_2$, 13.0 μg/$m^3$ for $NO_2$, and 46.3 μg/$m^3$ for $O_3$).

© 2019 Zhang C et al. *JAMA Network Open*.

**eTable 14.** Adjusted Odds Ratios (AORs) for Impaired Forced Vital Capacity and 4-Year Mean $PM_1$ Concentrations per 1–Interquartile Range Change (13.1 μg/$m^3$), Excluding Districts 1 at a Time Stratified by Breastfed Status

| Excluding district | Non-breastfed | Breastfed | Interaction |
|---|---|---|---|
| | ORs (95%CI) | ORs (95%CI) | *p* Value |
| Excluding district 1 | 2.67 (1.98-3.58) | 1.19 (0.96-1.47) | <0.001 |
| Excluding district 2 | 2.88 (2.13-3.89) | 1.26 (1.02-1.57) | <0.001 |
| Excluding district 3 | 2.65 (1.97-3.57) | 1.21 (0.98-1.50) | <0.001 |
| Excluding district 4 | 2.48 (1.86-3.31) | 1.16 (0.95-1.42) | <0.001 |
| Excluding district 5 | 2.67 (1.97-3.60) | 1.22 (0.98-1.51) | <0.001 |
| Excluding district 6 | 2.60 (1.93-3.51) | 1.20 (0.97-1.48) | <0.001 |
| Excluding district 7 | 2.79 (2.07-3.76) | 1.17 (0.94-1.45) | <0.001 |
| Excluding district 8 | 2.68 (2.00-3.60) | 1.20 (0.97-1.49) | <0.001 |
| Excluding district 9 | 2.71 (2.02-3.64) | 1.20 (0.97-1.49) | <0.001 |
| Excluding district 10 | 2.97 (2.19-4.03) | 1.21 (0.96-1.52) | <0.001 |
| Excluding district 11 | 2.78 (2.02-3.81) | 1.26 (1.00-1.59) | <0.001 |
| Excluding district 12 | 2.58 (1.91-3.50) | 1.10 (0.88-1.38) | <0.001 |
| Excluding district 13 | 2.68 (1.97-3.63) | 1.21 (0.96-1.51) | <0.001 |
| Excluding district 14 | 2.64 (1.96-3.55) | 1.23 (0.99-1.53) | <0.001 |
| Excluding district 15 | 2.68 (1.99-3.60) | 1.17 (0.94-1.45) | <0.001 |
| Excluding district 16 | 2.67 (1.98-3.59) | 1.17 (0.94-1.46) | <0.001 |
| Excluding district 17 | 2.81 (2.09-3.79) | 1.25 (1.01-1.55) | <0.001 |
| Excluding district 18 | 2.72 (2.01-3.69) | 1.15 (0.95-1.41) | <0.001 |
| Excluding district 19 | 2.97 (2.18-4.04) | 1.31 (1.05-1.63) | <0.001 |
| Excluding district 20 | 2.73 (2.02-3.68) | 1.19 (0.96-1.48) | <0.001 |
| Excluding district 21 | 2.49 (1.83-3.38) | 1.39 (1.11-1.74) | 0.002 |
| Excluding district 22 | 2.79 (2.07-3.77) | 1.18 (0.95-1.46) | <0.001 |
| Excluding district 23 | 2.82 (2.10-3.80) | 1.22 (0.98-1.51) | <0.001 |
| Excluding district 24 | 2.80 (2.08-3.76) | 1.21 (0.98-1.50) | <0.001 |

Abbreviations: CI, confidence interval; FVC, forced vital capacity; $PM_1$, airborne particulates with aerodynamic diameter <1 μm.

[a]Adjusted for age, sex, height, birth weight, preterm birth, parental education, family income per year, exercise per week, passive smoke exposure, home coal use, house pet, home renovation in the past 2 years, area of residence per person, doctor-diagnosed asthma, family history of atopy, and short-term air pollution concentrations.

© 2019 Zhang C et al. *JAMA Network Open*.

**eFigure 1.** Associations of the Prevalence of Impaired Lung Function With Ambient Pollutants Stratified by Breastfed Status

(A) for FVC and $PM_1$; (B) for $FEV_1$ and $PM_1$; (C) for PEF and $PM_1$; (D) for MMEF and $PM_1$; (E) for PEF and $PM_{2.5}$; (F) for MMEF and $PM_{2.5}$; (G) for PEF and $PM_{2.5}$; and (H) for MMEF and $PM_{2.5}$. The dose-response relationships of impaired lung function (%) with $PM_1$ and $PM_{2.5}$ estimated using a spatial statistical model were assessed at the study district level. The prevalence of impaired lung function (%) was adjusted for age, sex, height, birth weight, preterm birth, parental education, annual family income per year, exercise per week, passive smoke exposure, home coal use, house pet, home renovation in the past 2 years, area of residence per person, doctor-diagnosed asthma, family history of atopy, and short-term air pollution concentrations.

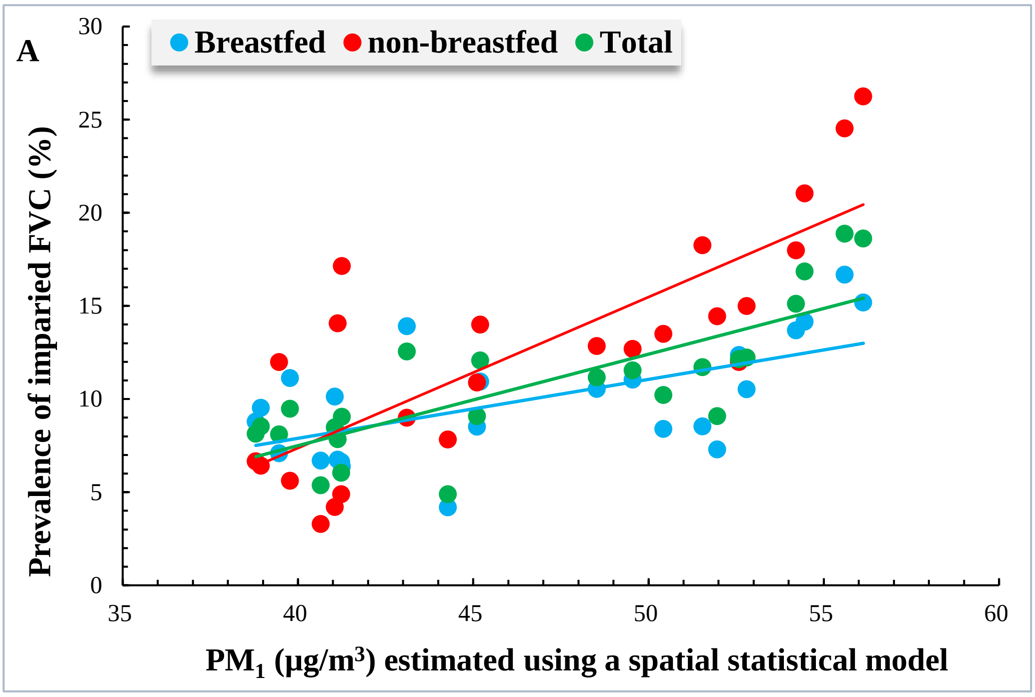


© 2019 Zhang C et al. *JAMA Network Open*.

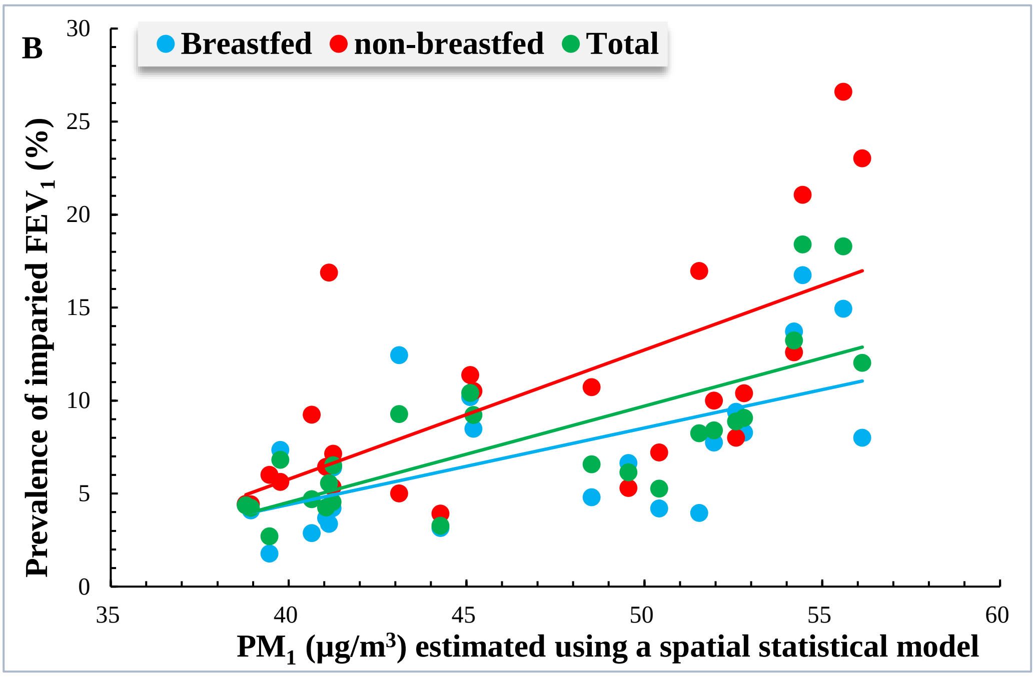


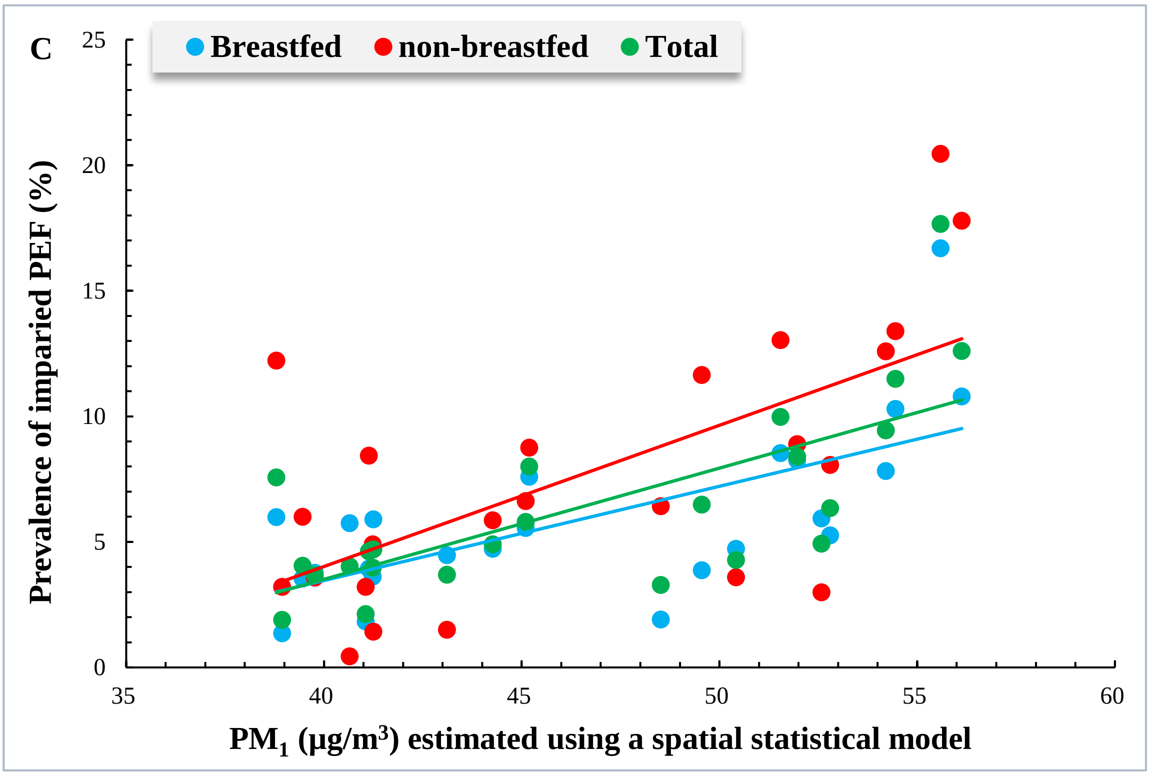


© 2019 Zhang C et al. *JAMA Network Open*.

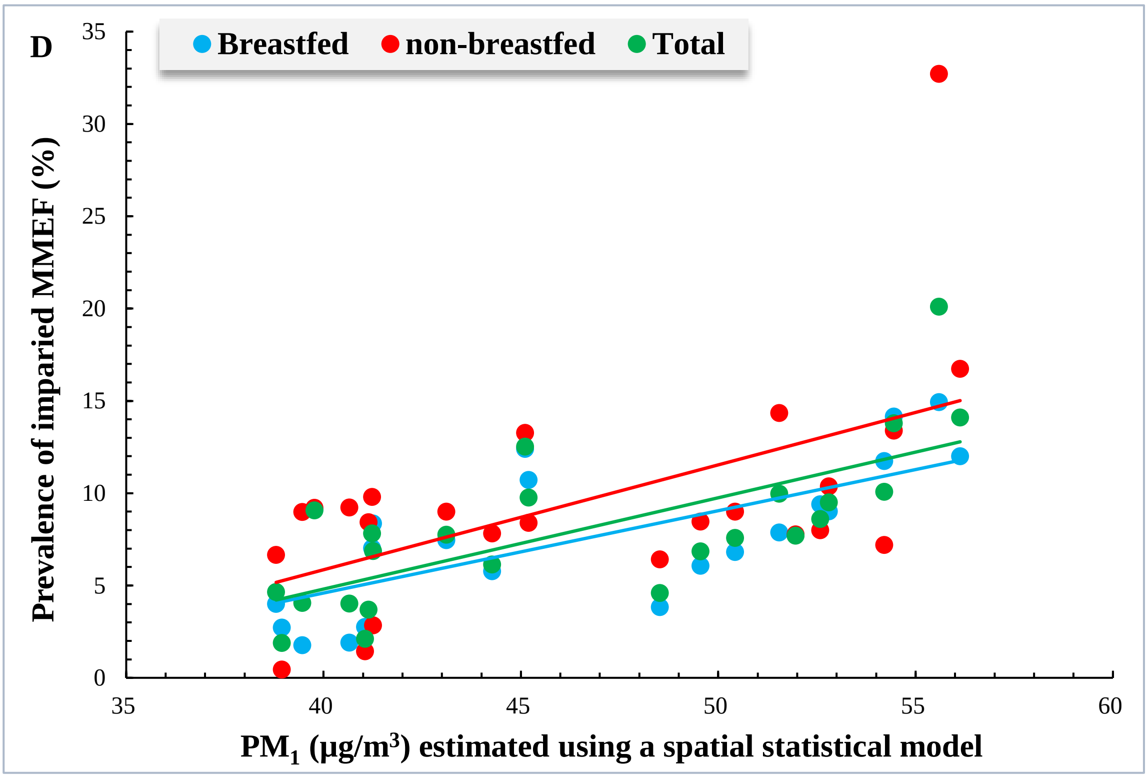


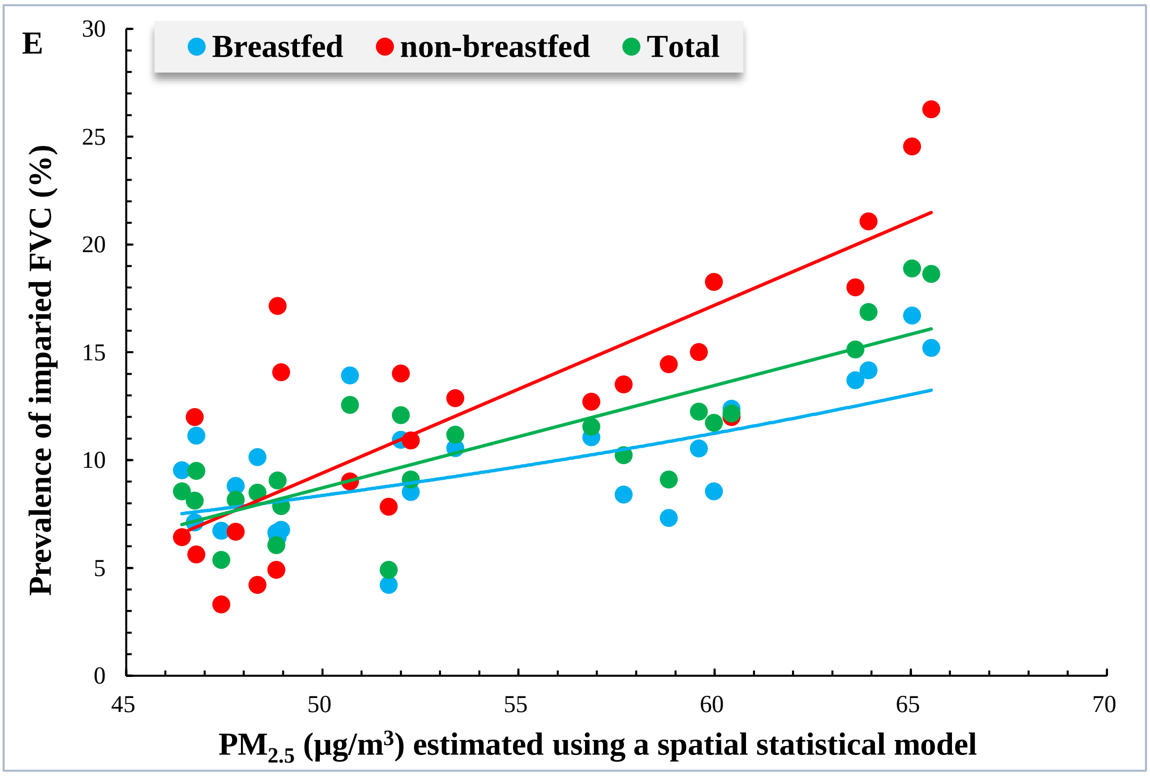


© 2019 Zhang C et al. *JAMA Network Open*.

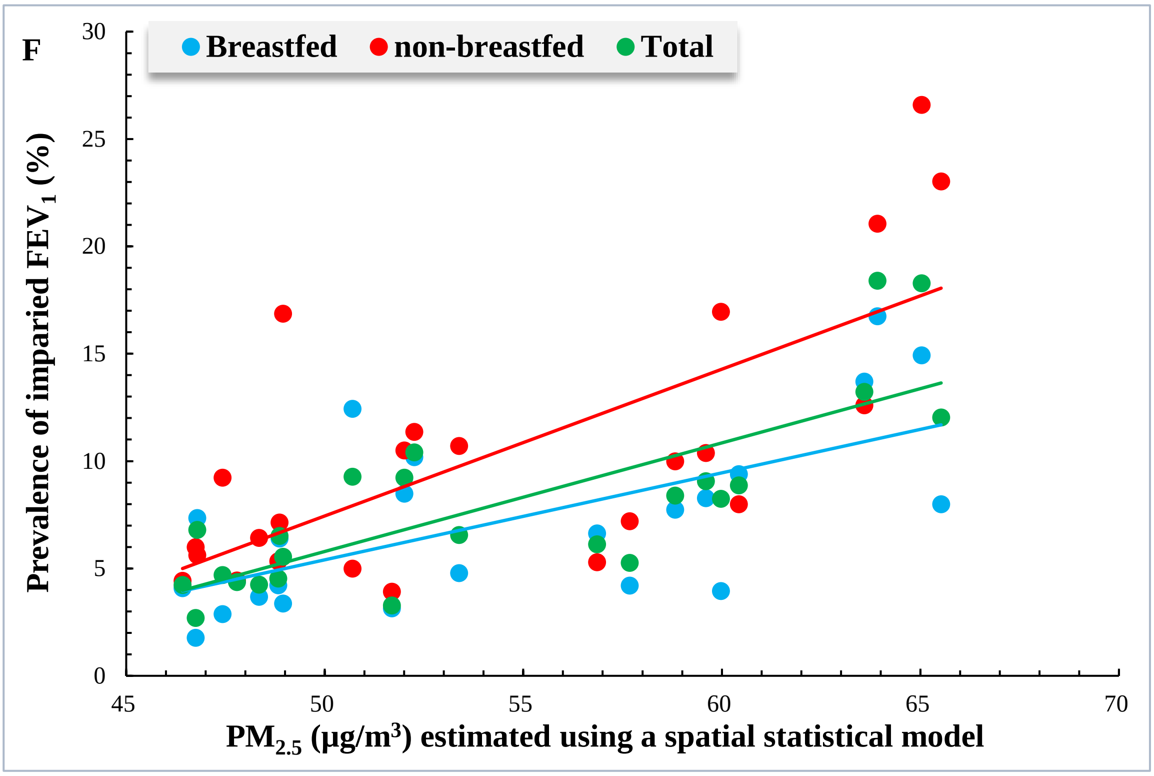


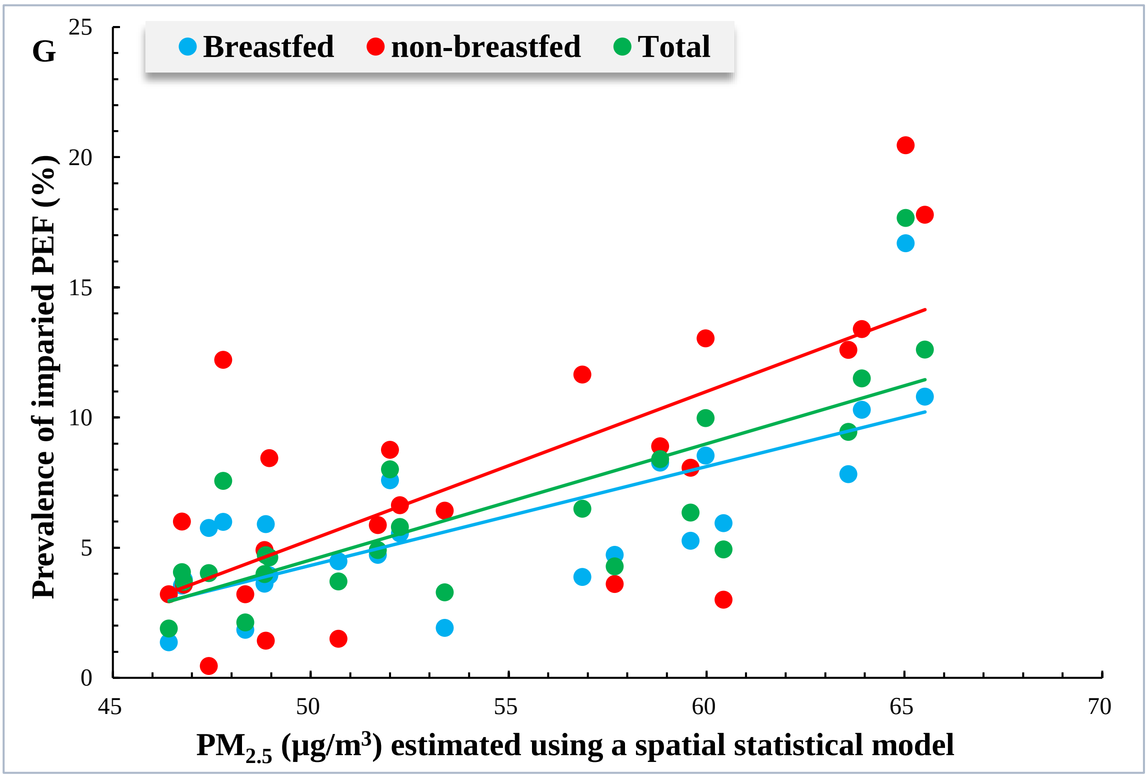


© 2019 Zhang C et al. *JAMA Network Open*.

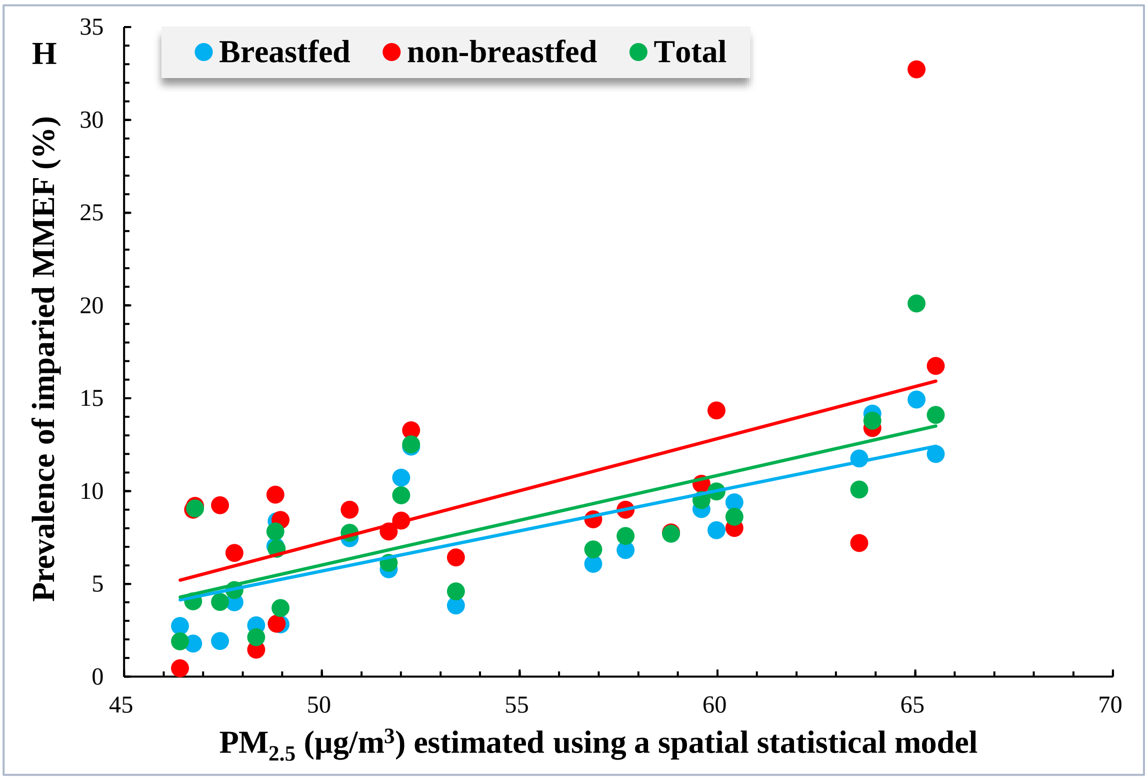


© 2019 Zhang C et al. *JAMA Network Open*.

**eFigure 2.** Estimated Absolute Change in Lung Function Test Measurements Associated With 4-Year Mean Ambient Air Pollutant Concentrations Estimated Using a Spatial Statistical Model Stratified by Breastfed Status

Adjusted for age, sex, height, birth weight, preterm birth, parental education, family income per year, exercise per week, passive smoke exposure, home coal use, house pet, home renovation in the past 2 years, area of residence per person, doctor-diagnosed asthma, family history of atopy, and short-term air pollution concentrations. Effect expressed for a 1 interquartile range (i.e., $75^{th}$ %tile - $25^{th}$ %tile) change in ambient concentration for each pollutant (13.1 μg/$m^3$ for $PM_1$, 10.0 μg/$m^3$ for $PM_{2.5}$).

© 2019 Zhang C et al. *JAMA Network Open*.

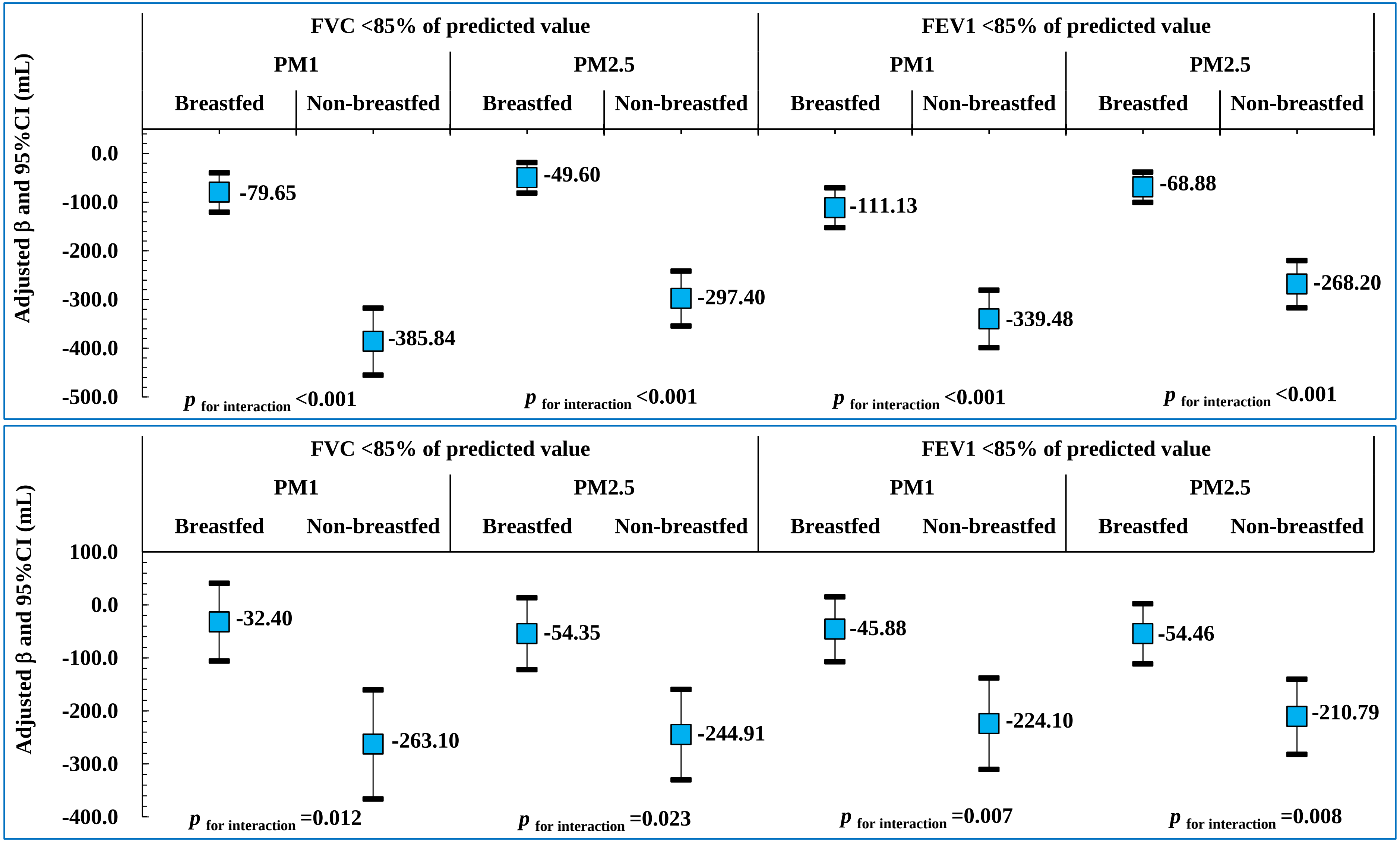


© 2019 Zhang C et al. *JAMA Network Open*.
